# Efficacy and safety of a self-applied carrageenan-based gel to prevent human papillomavirus infection in sexually active young women (CATCH study): an exploratory phase IIB randomised, placebo-controlled trial

**DOI:** 10.1016/j.eclinm.2023.102038

**Published:** 2023-06-08

**Authors:** Cassandra Laurie, Mariam El-Zein, Sarah Botting-Provost, Joseph E. Tota, Pierre-Paul Tellier, François Coutlée, Ann N. Burchell, Eduardo L. Franco

**Affiliations:** a Division of Cancer Epidemiology, McGill University, Montreal, Canada; b Epidemiology Department, Merck & Co. Inc., Rahway, NJ, USA; c Department of Family Medicine, McGill University, Montreal, Canada; d Laboratoire de Virologie Moléculaire, Centre de Recherche, Centre Hospitalier de l'Université de Montréal (CRCHUM), et Département de Microbiologie, Infectiologie et Immunologie, Université de Montréal, Montréal, Canada; e Department of Family and Community Medicine, and MAP Centre for Urban Health Solutions, Li Ka Shing Knowledge Institute, St. Michael's Hospital, Unity Health Toronto, Toronto, Canada

**Keywords:** Carrageenan, Gel, HPV, Human papillomavirus, Microbicide, Randomized controlled trial

## Abstract

**Background:**

Carrageenan demonstrated potent anti-HPV (human papillomavirus) activity *in vitro* and in animal models. The Carrageenan-gel Against Transmission of Cervical Human papillomavirus trial’s interim analysis (n = 277) demonstrated a 36% protective effect of carrageenan against incident HPV infections. Herein, we report the trial’s final results.

**Methods:**

In this exploratory phase IIB randomised, placebo-controlled trial, we recruited healthy women aged ≥18 years primarily from health service clinics at two Canadian Universities in Montreal. Participants were randomised (1:1) by the study coordinator (using computer-assisted block randomisation with randomly variable block sizes up to a block size of eight) to a carrageenan-based or placebo gel to be self-applied every other day for the first month and before/after intercourse. Participants, study nurses, and laboratory technicians (HPV testing and genotyping) were blinded to group assignment. At each visit (months 0, 0.5, 1, 3, 6, 9, 12), participants provided questionnaire data and a self-collected vaginal sample (tested for 36 HPV types, Linear Array). The primary outcome was type-specific HPV incidence (occurring at any follow-up visit). Intention-to-treat analyses for incidence were conducted using Cox proportional hazards regression models, including participants with ≥2 visits. Safety analyses included all participants randomised. This trial is registered with the ISRCTN registry, ISRCTN96104919.

**Findings:**

Between Jan 16, 2013 and Sept 30, 2020, 461 participants (enrolled) were randomly assigned to the carrageenan (n = 227) or placebo (n = 234) groups. Incidence and safety analyses included 429 and 461 participants, respectively. We found 51.9% (108/208) of participants in carrageenan and 66.5% (147/221) in placebo arm acquired ≥1 HPV type (hazard ratio 0.63 [95% CI: 0.49–0.81], p = 0.0003). Adverse events were reported by 34.8% (79/227) and 39.7% (93/234) of participants in carrageenan and placebo arm (p = 0.27), respectively.

**Interpretation:**

Consistent with the interim analysis, use of a carrageenan-based gel compared to placebo resulted in a 37% reduction in risk of incident genital HPV infections in women with no increase in adverse events. A carrageenan-based gel may complement HPV vaccination.

**Funding:**

10.13039/501100000024Canadian Institutes of Health Research, CarraShield Labs Inc.

**Research in context**

**Evidence before this study**

We searched PubMed using the keywords “carrageenan” and “human papillomavirus” or “HPV” from inception to January 9th, 2023, without applying language or date restrictions, to identify 23 experimental research articles that reported on carrageenan’s anti-HPV activity (carrageenan alone or in combination with other anti-microbial agents). Results were consistent with a protective effect of carrageenan in 1) pre-clinical studies (n = 18), 2) a post-hoc analysis showing lower HPV prevalence in the carrageenan group among the most compliant, 3) an observational study showing carrageenan may accelerate clearance of existing HPV infection, and 4) an interim analysis of the CATCH study demonstrating a 36% protective effect of carrageenan against incident HPV infection(s). Conversely, a clinical trial conducted in men who have sex with men did not demonstrate a protective effect of carrageenan on incidence, or clearance of anal HPV infections, and reported more adverse events in the carrageenan relative to the placebo arm.

**Added value of this study**

The CATCH study is the first clinical trial designed to assess the efficacy of a carrageenan-based gel in reducing the risk of incident and prevalent HPV infections in women. Results were consistent when considering HPV subgenera and type-specific analyses. The addition of carrageenan to a lubricant gel does not appear to impact gel tolerability.

**Implications of all the available evidence**

The results of the CATCH trial indicate that carrageenan-based gels could complement HPV vaccination in protecting against HPV-related diseases. Our findings of the clinical efficacy of carrageenan may encourage future research in this area. It would be important to further examine adherence by looking at determinants of adherence, explore the possibility of the addition of carrageenan to condoms, assess the impact of a carrageenan-based gel on anal HPV infections in women, and continue research in the area of multi-purpose prevention technology for agents against HPV, human immunodeficiency virus, and other sexually transmitted infections.

## Introduction

The most common sexually transmitted infection (STI) agent worldwide is human papillomavirus (HPV), which is a necessary cause of cervical cancer.^1^ Globally, cervical cancer is the fourth most commonly diagnosed cancer in women, with an estimated 604,127 new cases and 341,831 deaths in 2020.^2^ Elimination of cervical cancer is believed to be within our reach as per the World Health Organization’s (WHO) 2020 call to eliminate cervical cancer through a three-pronged approach: 1) HPV vaccination of 90% of girls by age 15, 2) cervical cancer screening of 70% of women aged 35–45, and 3) treatment of 90% of women with cervical precancers and cancers.^3^

While HPV vaccination is a highly effective intervention for primary prevention of HPV, the development, implementation, and maintenance of HPV vaccination programs across different settings are not without their challenges, including the negative impact of the COVID-19 pandemic derailing the uptake of HPV vaccines, temporary HPV vaccine shortage, cold chain requirements, and vaccine hesitancy.^4^ Although the global HPV vaccine supply is estimated to meet global demand,^5^ the HPV vaccine shortage highlights the need for the development of complementary primary prevention methods. The use of a personal self-administered gel may support the WHO’s goals to eliminate cervical cancer.

Carrageenan, an anionic polymer derived from red algae, previously showed promise as a potent anti-HPV inhibitor *in vitro* and *in vivo*,^6^ and has a good safety profile for vaginal use.^7^ The Carrageenan-gel Against Transmission of Cervical Human papillomavirus (CATCH) trial was specifically designed to evaluate the efficacy of a carrageenan-based gel against incident and prevalent HPV infections.^8^ Based on the interim analysis (June 2017, n = 277), carrageenan-gel use was associated with a 36% protective effect against incident HPV infections compared to placebo gel use.^9^ Anti-microbial agents, such as carrageenan, represent a promising area of research in STI prevention. We now report the results of the final analysis of efficacy and safety of a carrageenan-based gel in reducing HPV incidence and prevalence of genital HPV among sexually-active young women.

## Methods

### Study design

The CATCH trial is an exploratory phase IIB block-randomised, placebo-controlled trial. The full details of the study protocol (changes to the protocol are described in Supplementary Table S1) and data collection were previously described.^8^ We recruited female university students through McGill Health Service Clinic, Concordia Health Services, the Centre intégré de santé et de services sociaux de la Montérégie-Centre-Territoire Champlain-Charles-Le Moyne, and (as of September 2018) at the Gerald Bronfman Department of Oncology at the Division of Cancer Epidemiology of McGill University, Québec, Canada. Enrollment and follow-up visits were conducted at months 0.5, 1, 3, 6, 9, and 12. Study data were securely stored by LFC hosting until December 15, 2019. Thereafter, data were collected and stored using REDCap (Research Electronic Data Capture) tools.^10^, ^11^ The study received ethical approval from McGill University, Centre Hospitalier de l’Université de Montréal, Concordia University, and the Centre intégré de santé et de services sociaux de la Montérégie-Centre-Territoire Champlain-Charles-Le Moyne.

The trial findings are reported following the CONSORT (Consolidated Standards of Reporting Trials) 2010 checklist.^12^

### Participants

Eligible women to participate were those aged 18 years and older, living in Montreal and planning to remain in Montreal for at least the next year, who had vaginal sex with a male partner during the past 3 months and expect do so again in the next 3 months (regardless of whether or not the male partner(s) will change), not currently in a relationship that has lasted longer than 6 months (i.e., likely to be exposed to new HPV infections), willing to follow study instructions and attend follow-up visits for 12 months, who understand French or English, and who use a medically acceptable method of contraception and intend to use it for the duration of the trial. Women were considered ineligible based on the following exclusion criteria: have had a hysterectomy, have a history of cervical lesions/cancer or genital warts, pregnant or planning to immediately become pregnant, currently breast-feeding, had a recent (within the last 6 weeks) pregnancy, abortion, or genital surgery, have human immunodeficiency virus (HIV) infection, have a known allergy or hypersensitivity to vaginal lubricants and have allergy to any of the ingredients of the study product or placebo, have participated in any research studies (past 3 months) related to HPV or cervical cancer, or have participated in any research studies (past 3 months) that require taking medications or supplements, undergo medical tests or procedures, or undertake dietary or exercise regimens. Prior to randomisation, participants gave written informed consent (and e-consent as of September 5, 2019), which was administered by the study nurse.

### Randomisation and masking

Participants were randomised (1:1) to a carrageenan-based or placebo gel by the study coordinator, using computer-assisted block randomisation with randomly variable block sizes (up to a block size of 8). The intervention and placebo gels were packaged in identical containers, except for the product code label; four different codes were used for the intervention gel and an additional four for the control. The participant, study nurses, and laboratory technicians (HPV testing and genotyping) were blinded to group assignment.

### Procedures

Participants were instructed to apply the study gel every other day for the first month as well as before (and after as of October 26, 2015) each vaginal intercourse. Approximately 5–10 mL of the gel was to be applied to the vagina, penis, and/or condom. STI protection methods (e.g., condoms) were encouraged. Participants were asked to refrain from intercourse at least 48 h prior to their follow-up visits. Both study gels are commercially available (Divine9 and Divine), made by CarraShield Labs Inc (St Petersburg, FL). The study gels are identical; both are water-based, clear, odourless, tasteless, and of a similar viscosity. The distinguishing feature is the inclusion of carrageenan in the intervention gel.

At enrollment and each follow-up visit, participants completed a computer-assisted questionnaire on sociodemographic characteristics, smoking and alcohol use, medical and sexual history (only at enrollment), sexual activity and study gel use since last visit (only at follow-up), and condom and lubricant use (both enrollment and follow-up). In addition, a daily online calendar included questions on sexual behaviour, gel use, and adverse events (AEs). Participants could enter and modify calendar information for the seven previous days, which required them to log in at least once a week with a username and password. Additional details are provided in Supplementary Fig. S1 and Table S2A–S2C.

A self-collected vaginal sample was obtained at each study visit. Samples were tested using Linear Array (Roche Molecular Diagnostics, Branchburg, NJ) for detection and genotyping of 36 HPV types.^13^ Samples were tested for beta-globin prior to genotyping. Of 2510 samples, 19 were unavailable (12 samples were beta-globin negative and 7 were mishandled). Carrageenan as used in the clinical protocol above infrequently inhibited HPV detection in genital samples.^14^

### Outcomes

The primary outcome was presence of a newly detected vaginal HPV infection that was not detected at enrollment, and the secondary outcomes were HPV type-specific clearance of infection(s) detected at enrollment (i.e., reduction of HPV prevalence) and adherence (i.e., compliance) to the intervention measured using data from the daily online calendar. Adherent participants were defined as those whose cumulative adherence to gel use was as recommended in >50% of all intercourse acts prior to failure/censoring (Supplementary Table S1). We also assessed safety of the study gels by compiling all adverse events that were reported.

### Statistical analysis

The calculation of sample size was informed by two cohort studies conducted in Montreal based on a cumulative incidence proportion of infection at 1 year of 19.5% and a clearance rate of 38% after 1 year. Assuming an expected effect size of 50%, we calculated a sample size of 463 for incidence and 388 for clearance, which accounted for 10% loss to follow-up, a 2-sided hypothesis with 80% power and type I error of 0.05.^8^

Based on oncogenic risk and tissue tropism, we grouped HPV types into subgenera; low-risk types in subgenus 1: HPVs 6, 11, 40, 42, 44, and 54; high-risk types in subgenus 2: HPVs 16, 18, 26, 31, 33, 34, 35, 39, 45, 51, 52, 53, 56, 58, 59, 66, 67, 68, 69, 70, 73, and 82; and commensal HPVs in subgenus 3: HPVs 61, 62, 71, 72, 81, 83, 84, and 89.^15^, ^16^, ^17^ Baseline visits with missing HPV data (n = 3) were imputed based on data at the second visit (on average, 14 days after the first visit).

Adherence to the intervention was defined as the number of gel uses divided by the number of vaginal intercourses in the seven days prior to the study visit (from follow-up survey data) and between consecutive study visits (from calendar data). We compared adherence between groups and study visits. Due to (non-significant) imbalances in baseline HPV vaccination status between arms and discrepancies in reporting of vaccination status between screening (pre-enrollment) and the baseline survey (at enrollment), we validated participants’ vaccination status by either confirming their status via email or (in the case of no email reply) by using the date of vaccination reported in their baseline or follow-up survey (Supplementary Fig. S2 and Table S3A–S3C).

Cox proportional hazards regression models were used to evaluate the efficacy of carrageenan in reducing vaginal HPV incidence/detection and prevalence, according to an intention-to-treat (ITT) approach. The analysed population consisted of a subset of the ITT population, as it was not possible to include participants without follow-up visits in the analysis. The proportional hazards assumption was assessed based on Schoenfeld residuals and suggested no violation of the assumption (p = 0.39). Start time was set to the date of the baseline visit when participants were randomised and the first sample was collected. The end time was the date the participant was either censored (due to a participant completing participation or withdrawing from the study) or had the outcome. Missing HPV data for follow-up visits were censored in incidence and clearance analyses. Incidence analyses, performed by any HPV positivity (if a participant was positive for one or more HPV types at baseline, they remain at risk of acquiring HPV types absent at baseline) and subgenera (separate analyses done for each of the three subgenera), included participants who had at least 1 follow-up visit. We performed exploratory sub-group analyses to compare the intervention effects according to main baseline characteristics and cumulative adherence to gel use prior to failure (new detection of an HPV type) or censoring and whether participants were enrolled prior or after amendment to gel usage instructions, i.e., gel use before intercourse vs. before and after intercourse.

We performed additional post-hoc sensitivity analyses to account for missing outcome data over follow-up. First, we performed a complete-case analysis restricting to participants with valid HPV results at all study visits. Second, we applied single imputation methods involving 1) a best-worst analysis which assumed all participants with missing HPV data in the carrageenan arm were HPV-negative over follow-up and those in the placebo arm had a newly detected HPV infection, and 2) a worst-best analysis which assumed all participants with missing HPV data in the carrageenan arm had a newly detected HPV infection and those in the placebo arm were HPV-negative over follow-up.^18^ Finally, we used inverse probability weighting (IPW) to account for missing outcome data by using logistic regression to predict the probability of being a complete case, calculating the inverse of the probability for being a complete case, and applying these weights in a Cox regression model to obtain an effect estimate in the weighted population. To account for slight baseline imbalances and adherence, we included a model adjusted for baseline variables (HPV prevalence, age at first sexual intercourse, partners in the last month), and adherence in the past 7 days.

Participants were included in clearance analyses if they had at least two study visits and were HPV-positive at baseline. We considered two definitions of clearance: liberal (one HPV-negative visit following at least one HPV-positive visit) and conservative (two consecutive HPV-negative visits following at least one HPV-positive visit). We considered time to 1) clearance of all baseline HPV infections and 2) first clearance of any baseline HPV infection.

Our primary analyses for incidence and clearance were also done at the HPV-level, where the unit of analysis was each individual HPV type. The Cox proportional hazards models were stratified by HPV type and clustered by participant. For incidence analyses, participants were considered at risk for any HPV type absent at baseline, where each participant could contribute up to 36 observations, each corresponding to an HPV type. For HPV-level clearance analyses, participants were considered at risk for clearing any of their baseline HPV infections.

To assess safety of the study gels, we tabulated self-reported AEs and/or reactions that were recorded by means of the 1) *daily calendar*, 2) *follow-up survey* administered at each follow-up visit, 3) *adverse event module (nurse report)* during follow-up visits, and/or 4) reporting of an adverse event during *adverse event follow-up*. AEs reported in the daily calendar were graded (mild, moderate, or severe) by participants.

Data management and analyses were done in SAS V9.4 and Stata version 17, respectively. The trial was registered (ISRCTN96104919) and approved by Health Canada (authorisation file number 169160). A data safety and monitoring board reviewed and recommended publication of the previously published interim analysis,^9^ as well as the final trial results reported herein.

### Role of funding source

The funders of the study had no role in study design, data collection, data analysis, data interpretation, or writing of the report. All authors had access to the dataset and had final responsibility for the decision to submit for publication.

## Results

**Fig. 1:**
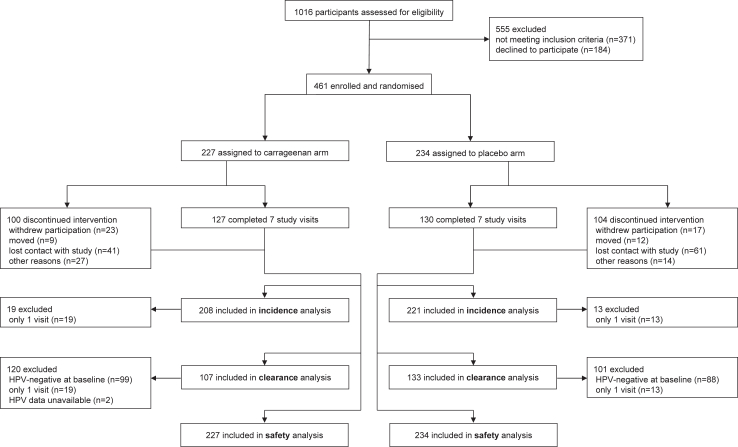
**Trial profile: design and subject allocation.** The CONSORT flow diagram displays the total number of participants assessed for eligibility who were subsequently enrolled and randomised into either the carrageenan or placebo arm. Reasons for exclusion and discontinuing the intervention are provided. A total of 429 participants were included in the incidence analyses, 240 in the clearance analyses, and 461 in the safety analysis.

Recruitment took place between January 16th, 2013 and October 30th, 2020. As shown in Fig. 1, 1016 participants were assessed for eligibility. Of these, 461 were randomised into the carrageenan (n = 227) and placebo (n = 234) arms. Study visits 2–7 were completed by 461, 429, 399, 362, 318, 291, and 257 participants, respectively. Overall, 429, 240, and 461 participants were included in the incidence, clearance, and safety analyses, respectively. Supplementary Fig. S3 shows the distribution of time between baseline and subsequent visits compared to the study schedule. While there were variations between the actual and planned study schedule, little difference was observed between intervention arms.

Baseline characteristics of study participants are shown in Table 1. The median age was 23.0 in the carrageenan and 21.9 in the placebo arm. Most participants were Canadian, single, never smokers, had less than 5 lifetime sexual partners, and had 1 sexual partner in the past month. The median age at first intercourse was 17. More participants in placebo (60.3%, 141/234) compared to carrageenan (51.3%, 115/224) arm were infected with any HPV type at baseline, which was consistent by subgenera. Detailed HPV prevalence data at each study visit are shown for carrageenan and placebo arms in Supplementary Table S4A and S4B, respectively. Fewer participants in the carrageenan (26.1%, 54/207) compared to placebo arm (31.7%, 70/221) reported ever having anal intercourse during follow-up. Fewer participants in carrageenan (42.7%, 97/227) compared to placebo (51.3%, 120/234) arm reported being vaccinated against HPV. The difference in vaccination status between arms was smaller based on validated vaccination status; 42.3% (47/111) of participants in carrageenan and 49.2% (61/124) in placebo arm reported receiving the HPV vaccine.

**Table 1:** Baseline characteristics of participants, by study arm.

	Carrageenan (n = 227)	Placebo (n = 234)
**Age—years**		
Median (IQR)	23.0 (20.5–27.8)	21.9 (19.8–25.1)
Range	18.2–68.5	18.0–53.0
**Ethnicity, n (%)**		
French Canadian	53 (23.4)	47 (20.1)
English Canadian	62 (27.3)	68 (29.1)
Black Canadian	14 (6.2)	15 (6.4)
Latin American	22 (9.7)	22 (9.4)
South Asian	9 (4.0)	7 (3.0)
East Asian	16 (7.1)	24 (10.3)
Other or not reported	51 (22.5)	51 (21.8)
**Marital status, n (%)**		
Single	145 (63.9)	160 (68.4)
Living with a partner or married	22 (9.7)	23 (9.8)
Divorced, separated, or widowed	60 (26.4)	51 (21.8)
**Smoking status, n (%)**^a^		
Never	157 (69.2)	147 (62.8)
Former	50 (22.0)	59 (25.2)
Current	19 (8.4)	27 (11.5)
**Age at first intercourse, years**^a^		
Median (IQR)	17 (16–19)	17 (16–18)
Range	9–36	12–32
Not reported, n	5	5
**Lifetime sex partners—quantiles, n (%)**		
<5	61 (26.9)	60 (25.6)
5–7	43 (18.9)	35 (15.0)
8–11	37 (16.3)	47 (20.1)
12–20	47 (20.7)	50 (21.4)
≥21	39 (17.2)	42 (18.0)
**No. of sex partners in the past month, n (%)**^a^		
0	42 (18.5)	36 (15.5)
1	144 (63.4)	144 (61.8)
≥2	41 (18.1)	53 (22.8)
**Anal intercourse in the past month, n (%)**^a^		
Yes	25 (11.0)	26 (11.2)
**HPV DNA status, n (%)**		
Any HPV^b^	115 (50.7)	141 (60.3)
Negative	109 (48.0)	93 (39.7)
Missing PCR results^c^	3 (1.32)	0 (0)
Subgenus 1^d^	38 (17.0)	55 (23.5)
Subgenus 2^e^	96 (42.9)	114 (48.7)
Subgenus 3^f^	60 (26.8)	76 (32.5)
**HPV vaccination status, n (%)**		
Yes	97 (42.7)	120 (51.3)
**Validated HPV vaccination status**^g^**, n (%)**		
Yes	47 (20.7)	61 (26.1)
No	64 (28.2)	63 (26.9)
Missing	116 (51.1)	110 (47.0)

Race categories with fewer than 5 participants in each group were collapsed to respect participants anonymity.

n: number of participants, SD: standard deviation, IQR: interquartile range, PCR: polymerase chain reaction, HPV: human papillomavirus, DNA: deoxyribonucleic acid.

a Data were not reported by 2, 10, 1, and 2 participants, respectively.

b Participant tested positive for at least 1 of 36 HPV types.

c Missing results correspond to invalid or mishandled samples.

d Subgenus 1 group includes HPVs 6, 11, 40, 42, 44, and 54.

e Subgenus 2 group includes HPVs 16, 18, 26, 31, 33, 34, 35, 39, 45, 51, 52, 53, 56, 58, 59, 66, 67, 68, 69, 70, 73, and 82.

f Subgenus 3 group includes HPVs 61, 62, 71, 72, 81, 83, 84, and 89.

g Details of the validation of participants’ vaccination status can be found in the Supplementary Fig. S2 and Table S3A–S3C).

Follow-up characteristics of the participants are shown in Supplementary Table S5. The median overall follow-up time was 12.2 months. At the observation level (observations between two consecutive study visits), adherence was comparable between arms; 27.5% (272/988) of participants in carrageenan and 26.4% (282/1068) in placebo arm reported >75% adherence in the 7 days preceding each visit based on data from the follow-up surveys.

**Fig. 2:**
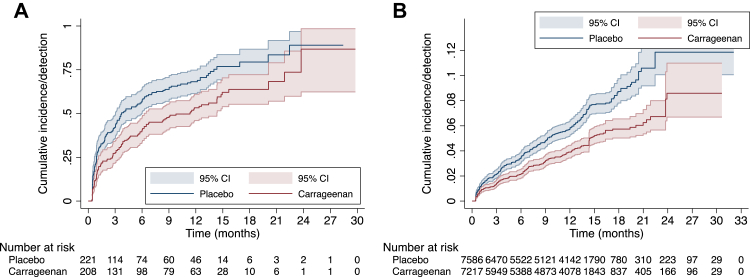
**Cumulative incidence/detection of HPV at the participant- and HPV-level.** The plots represent Kaplan–Meier failure functions. Fig. 2A plots the first HPV infection episode detected using women as the unit of observation (i.e., participant-level analysis); the number at risk corresponds to the number of women who were HPV-negative for at least 1 of the 36 HPV types at baseline. The result of the log-rank test was p = 0.0002. Fig. 2B plots all new HPV infections based on HPV-level analysis; the number at risk corresponds to the number of infections a participant could have acquired at subsequent visits. Each participant could have acquired any of the 36 HPV types for which they were negative at baseline. Confidence bands are based on log transformation. CI: confidence interval.

As shown in Table 2, fewer participants in carrageenan (51.9%, 108/208) acquired an incident HPV infection compared to placebo (66.5%, 147/221) arm (HR 0.63, 95% CI 0.49–0.81). This effect was consistently observed in participant-level analyses by subgenera (HRs ranged from 0.58–0.70) and in the HPV-level analysis (HR 0.65, 95% CI 0.50–0.83). Fig. 2 shows the cumulative incidence of HPV by intervention arm; participant- (Fig. 2A) and HPV- (Fig. 2B) level results were consistent with a protective effect of carrageenan against incident HPV infections. The estimates from the Kaplan–Meier survivor function are shown in Supplementary Table S6. Results from all sensitivity analyses (complete case, best-worst scenario, worst-best, imbalance adjusted, and IPW) were consistent with a protective effect of carrageenan; the HRs (and corresponding CIs) were 0.64 (0.49–0.82), 0.62 (0.49–0.80), 0.71 (0.55–0.91), 0.66 (0.51–0.85), and 0.61 (0.47–0.79), respectively (Supplementary Table S7).

**Table 2:** Incidence of any HPV infection and grouped infections at the participant- and HPV-level, by study arm.

Analysis level	HPV infection grouping	Carrageenan				Placebo				Effect estimate	
		N incident/N at risk (%)	Actuarial mean^a^ (95% CI)	Arithmetic mean^a^ (95% CI)	Median^a^ (95% CI)	N Incident/N at risk (%)	Actuarial mean^a^ (95% CI)	Arithmetic mean^a^ (95% CI)	Median^a^ (95% CI)	Hazard ratio (95% CI)	p-value
Participant	Any HPV	108/208 (51.9)	12.6^b^ (10.6–14.7)	4.8 (3.8–5.7)	11.3 (6.2–14.3)	147/221 (66.5)	8.7^b^ (7.1–10.3)	3.5 (2.8–4.2)	3.7 (3.0–6.0)	0.63 (0.49–0.81)	0.0003
	Subgenus 1^c^	33/208 (15.9)	24.8^b^ (22.9–26.7)	6.6 (4.8-8.3)	NR	56/221 (25.3)	22.7^b^ (20.5–24.8)	5.3 (4.0–6.6)	NR (17.8-)^d^	0.58 (0.38–0.89)	0.011
	Subgenus 2^e^	84/208 (40.4)	16.1^b^ (13.8–18.4)	5.0 (3.8–6.1)	14.9 (12.6–)^d^	124/221 (56.1)	11.1 (9.3–12.9)	4.4 (3.6–5.3)	8.6 (6.0–12.0)	0.61 (0.46–0.81)	0.0004
	Subgenus 3^f^	55/208 (26.4)	20.7^b^ (18.3–23.1)	5.7 (4.2–7.3)	23.7 (20.1–)^d^	79/221 (35.8)	19.2^b^ (16.8–21.6)	5.5 (4.4–6.5)	22.4 (13.6-)^d^	0.70 (0.50–0.99)	0.040
HPV	Any HPV^g^	278/7217 (3.9)	29.1^b^ (28.9–29.3)	6.7 (6.0–7.3)	NR	438/7586 (5.8)	29.7^b^ (29.4–29.9)	6.4 (5.9–6.9)	NR	0.65 (0.50–0.83)	0.0005

CI: confidence interval, HPV: human papillomavirus, NR: not reached, N: number.

a Follow-up time in months. The calculation of the median and actuarial mean follow-up time included all participants, whereas the arithmetic mean restricted to participants who acquired a new HPV type.

b Mean was underestimated since the largest observed analysis time was censored.

c Subgenus 1 includes HPVs 6, 11, 40, 42, 44, and 54.

d Upper confidence limit was undetermined since the survival function did not fall below 0.5.

e Subgenus 2 includes HPVs 16, 18, 26, 31, 33, 34, 35, 39, 45, 51, 52, 53, 56, 58, 59, 66, 67, 68, 69, 70, 73, and 82.

f Subgenus 3 includes HPVs 61, 62, 71, 72, 81, 83, 84, and 89.

g Proportional hazards Cox regression models account for all incident HPV infections acquired over follow-up. Participants were considered at risk for any HPV type absent at baseline. Each participant could contribute up to 36 observations, each corresponding to an HPV type. The unit of analysis was each individual HPV type.

Table 3 shows time to clearance of all HPV types and time to clearance of the first cleared HPV infection by clearance definition (liberal and conservative). The effect estimates were consistently greater than the null value, however, their respective 95% CIs consistently included the null value, irrespective of the analysis level, clearance outcome, or clearance definition. Based on liberal clearance definition of all HPV types, 57.9% (62/107) of participants cleared all their baseline infections in carrageenan and 49.6% (66/133) in placebo arm, corresponding to a HR of 1.41 (95% CI 0.99–2.00). By applying the conservative definition, the HR was 1.16 (95% CI 0.73–1.84). When considering only the first cleared infection, 79.4% (85/107) cleared at least 1 HPV infection in the carrageenan and 78.9% (105/133) in the placebo arm (HR 1.03, 95% CI 0.77–1.38) for the liberal definition; these values were 62.6% (67/107) and 60.9% (81/133) (HR 1.04, 95% CI 0.75–1.44) for conservative clearance, respectively. No remarkable differences were observed when considering time to clearance of individual HPV types; 66.8% (181/271) cleared at least 1 HPV type in the carrageenan and 65.7% (243/370) in the placebo arm (HR 1.17, 95% CI 0.90–1.51) for the liberal definition, and 45.0% (122/271) cleared at least 1 HPV type in the carrageenan and 45.7% (169/370) in the placebo arm (HR 1.10, 95% CI 0.84–1.43) for the conservative definition.

**Table 3:** Clearance, according to outcome and definition, of any HPV infection at the participant- and HPV-level, by study arm.

Analysis level	Outcome	Clearance definition^a^	Carrageenan				Placebo				Effect estimate	
			N cleared/N at risk (%)	Actuarial mean^b^ (95% CI)	Arithmetic mean^b^ (95% CI)	Median^b^ (95% CI)	N cleared/N at risk (%)	Actuarial mean^b^ (95% CI)	Arithmetic mean^b^ (95% CI)	Median^b^ (95% CI)	Hazard ratio (95% CI)	p-value
Participant	Time to clearance of all HPV types^c^	Liberal	62/107 (57.9)	10.0 (8.4–11.6)	5.4 (4.8–5.9)	9.3 (6.4–11.0)	66/133 (49.6)	13.0 (10.9–15.0)	5.8 (5.2–6.4)	11.1 (9.5–13.4)	1.41 (0.99–2.00)	0.055
		Conservative	34/107 (31.8)	14.8^d^ (13.0–16.6)	5.7 (5.0–6.5)	16.0 (10.5-NR)	39/133 (29.3)	19.3 (16.8–21.7)	6.2 (5.4–7.0)	28.9 (13.6-NR)	1.16 (0.73–1.84)	0.54
	Time to first cleared HPV infection^e^	Liberal	85/107 (79.4)	4.7^d^ (3.6–5.8)	5.0 (4.6–5.5)	3.0 (1.2–4.1)	105/133 (79.0)	4.8 (3.9–5.7)	5.4 (4.9–5.8)	3.2 (1.6–4.0)	1.03 (0.77–1.38)	0.83
		Conservative	67/107 (62.6)	6.8^d^ (5.4–8.1)	5.4 (4.9–6.0)	6.0 (4.1–6.5)	81/133 (60.9)	7.6^d^ (6.1–9.2)	5.7 (5.2–6.2)	5.8 (3.2–6.9)	1.04 (0.75–1.44)	0.81
HPV	Time to clearance of an individual HPV type^f^	Liberal	181/271 (66.8)	0.24 (0.2–0.3)	5.0 (4.2–5.7)	0.20 (0.1–0.2)	243/370 (65.7)	0.29 (0.26–0.32)	5.9 (5.3–6.5)	0.23 (0.21–0.25)	1.17 (0.90–1.51)	0.24
		Conservative	122/271 (45.0)	0.38^d^ (0.3–0.4)	5.1 (4.4–5.9)	0.31 (0.3–0.4)	169/370 (45.7)	0.45 (0.40–0.50)	5.6 (4.8–6.3)	0.34 (0.27–0.38)	1.10 (0.84–1.43)	0.50

CI: confidence interval, HPV: human papillomavirus, NR: not reached, N: number.

a Liberal clearance was defined as having a single HPV-negative visit following ≥1 HPV-positive visit(s). Conservative clearance was defined as having ≥2 consecutive HPV-negative visits following ≥1 HPV-positive visit(s).

b Follow-up time in months. The calculation of the median and actuarial mean follow-up time included all participants, whereas the arithmetic mean restricted to participants who acquired a new HPV type.

c Time to clearance of all baseline HPV infections (i.e., clearance was considered to have occurred once all baseline HPV infections cleared).

d Mean was underestimated since the largest observed analysis time was censored.

e Time to clearance of the first baseline HPV infection (i.e., clearance was considered to have occurred once the first of any baseline HPV infections cleared).

f Proportional hazards Cox regression models account for all baseline HPV types that cleared over follow-up. Participants were considered at risk for clearing any HPV type present at baseline. Each participant could contribute up to 36 observations, each corresponding to an HPV type. The unit of analysis was each individual HPV type.

Overall, there was a similar proportion of AEs reported in each arm: 34.8% (79/227) in the carrageenan and 39.7% (93/234) in the placebo arm (Table 4). Those most frequently reported were vaginal yeast infection, itching, burning or pain in the genital area, and bacterial vaginosis. Of participants who reported an AE in the daily calendar, 24.3% (9/37) in carrageenan and 30.8% (12/39) in placebo arm graded their AEs as severe (Supplementary Table S8); the most common severe AE was “itching, burning or pain in the genital area”. We did not observe a higher proportion of withdrawals among participants who reported adverse events nor among participants who reported difficulties using the study gel (Supplementary Table S9). The proportion of participants reporting difficulties with the study gel before, during, or after intercourse was similar between arms (77.9% [159/204] in the carrageenan and 80.7% [176/218] in the placebo arm); the most frequently reported difficulties were not having the CATCH gel at the time of intercourse or forgetting to use the CATCH gel (Supplementary Table S10).

**Table 4:** Adverse events [n (%)] reported through different sources, overall and by study arm.

	Overall (n = 461)	Carrageenan (n = 227)	Placebo (n = 234)	+ (more AE in CG arm)
**Any adverse event reported, overall**^a^	**172 (37.3)**	**79 (34.8)**	**93 (39.7)**	
**1. Daily calendar**^b^	**76 (17.6)**	**37 (17.8)**	**39 (17.3)**	
Unusually heavy or painful period	9 (2.1)	1 (0.5)	8 (3.6)	
Vaginal bleeding in between menstrual periods	14 (3.2)	6 (2.9)	8 (3.6)	
Pain during vaginal sex^c^	19 (4.8)	13 (6.9)	6 (2.9)	+
Unusual vaginal discharge	15 (3.5)	11 (5.3)	4 (1.8)	+
Itching, burning, or pain in the genital area	42 (9.7)	23 (11.1)	19 (8.4)	+
Genital sore/ulcer	5 (1.2)	2 (1.0)	3 (1.3)	
Needing to urinate more often than usual	10 (2.3)	3 (1.4)	7 (3.1)	
Pain while urinating	9 (2.1)	6 (2.9)	3 (1.3)	+
Blood in urine	4 (0.9)	3 (1.4)	1 (0.4)	+
Lower abdominal pain	8 (1.9)	5 (2.4)	3 (1.3)	+
Lower back pain not caused by physical exertion	2 (0.5)	0 (0)	2 (0.9)	
Other^d^	42 (9.7)	21 (10.1)	21 (9.3)	+
**2.1 Follow-up survey**^e^	**55 (13.0)**	**24 (11.7)**	**31 (14.2)**	
Gel use caused discomfort/adverse reactions to participant	49 (11.6)	22 (10.7)	27 (12.4)	
Gel use caused discomfort/adverse reactions to partner	10 (2.4)	5 (2.4)	5 (2.3)	+
**2.2 Follow-up survey, conditions**^f^	**91 (21.3)**	**43 (20.7)**	**48 (21.8)**	
Vaginal yeast infection	70 (16.5)	35 (17.0)	35 (16.0)	+
Trichomonas vaginal infection	6 (1.4)	3 (1.5)	3 (1.4)	+
Venereal warts, condyloma, or HPV	10 (2.3)	5 (2.4)	5 (2.3)	+
Chlamydia	12 (2.8)	6 (2.9)	6 (2.7)	+
Genital herpes	8 (1.9)	3 (1.4)	5 (2.3)	
Syphilis	4 (0.9)	2 (1.0)	2 (0.9)	+
Gonorrhea	4 (0.9)	2 (1.0)	2 (0.9)	+
Ulcers or genital sores	5 (1.2)	2 (1.0)	3 (1.4)	
Human immunodeficiency virus	4 (0.9)	2 (1.0)	2 (0.9)	+
Hepatitis B	4 (0.9)	2 (1.0)	2 (0.9)	+
Bacterial vaginosis	24 (5.6)	11 (5.3)	13 (5.9)	
**3. Adverse event module (nurse report)**^g^	**75 (16.3)**	**38 (16.7)**	**37 (15.8)**	+
Unusually heavy or painful period	6 (1.3)	1 (0.4)	5 (2.1)	
Vaginal bleeding in between menstrual periods	13 (2.8)	5 (2.2)	8 (3.4)	
Pain during vaginal sex	18 (3.9)	12 (5.3)	6 (2.6)	+
Unusual vaginal discharge	14 (3.0)	10 (4.4)	4 (1.7)	+
Itching, burning, or pain in the genital area	43 (9.3)	24 (10.6)	19 (8.1)	+
Genital sore/ulcer	5 (1.1)	2 (0.9)	3 (1.3)	
Needing to urinate more often than usual	8 (1.7)	3 (1.3)	5 (2.1)	
Pain while urinating	9 (2.0)	6 (2.6)	3 (1.3)	+
Blood in urine	4 (0.9)	3 (1.3)	1 (0.4)	+
Lower abdominal pain	6 (1.3)	4 (1.8)	2 (0.9)	+
Lower back pain not caused by physical exertion	2 (0.4)	0 (0)	2 (0.9)	
Other^d^	25 (5.4)	11 (4.9)	14 (6.0)	
**4. Adverse event follow-up**^g^	**20 (4.3)**	**9 (4.0)**	**11 (4.7)**	

The percentage of adverse events was calculated as the number of participants affected by an AE divided by the number of participants who ever responded to the question.

N: number of participants affected, +: a greater proportion of adverse events were reported in the carrageenan arm relative to the placebo arm, AE: adverse event, HPV: human papillomavirus.

a 15 participants in the carrageenan and 5 participants in the placebo arm never provided any information about adverse events. These participants only had 1 visit. However, these 20 participants were included in the calculation of any adverse reported overall, as for two sources (Adverse event module [nurse report] and (Adverse event follow-up), due to the nature of reporting, we used an inferred denominator that included all participants randomized.

b 28 participants were not included: 19 participants in the carrageenan arm and 9 participants in the placebo group never filled out the calendar.

c 63 participants were not included: 19 participants in the carrageenan and 16 participants in the placebo arm did not report intercourse, and 19 participants in the carrageenan arm and 9 participants in the placebo group never filled out the calendar.

d Other AEs reported included burning, irritation, urinary tract infection, spotting, and yeast infection among others.

e 37 participants were not included: 32 participants did not have a follow-up visit (19 in the carrageenan and 13 in the placebo group) and 5 participants (2 in the carrageenan arm and 3 in the placebo arm) did not respond to these questions in the follow-up survey.

f 33 participants were not included overall: 32 participants did not have a follow-up visit (19 in the carrageenan and 13 in the placebo group) and 1 participant (1 in the placebo group) did not respond to any of these questions in the follow-up survey.

g Due to the nature of reporting we used an inferred denominator, which included all participants randomized.

No differences were observed in sub-group analyses by baseline characteristics (age, ethnicity, marital status, smoking status, age at first intercourse, lifetime sexual partners, sex partners in the last month, HPV status at baseline, and vaccination status) and cumulative adherence to gel use; amendment to the gel use instruction (before intercourse only vs. before and after intercourse) appeared to result in a more protective effect compared to gel use before (HR 0.65 vs. HR 0.94 (Supplementary Fig. S4). There was also no evidence of a dose-response relationship based on cumulative adherence to gel use prior to failure or censoring, irrespective of data used (calendar data or follow-up survey data) nor categorisation (binary [<50, ≥50] or categorical), nor was a more protective effect observed when analyses were restricted to participants with an overall adherence >50% (per-protocol analysis, which consisted of a sub-set of the per-protocol population, excluding participants who did not report gel use and/or vaginal intercourse prior to failure or censoring) [Supplementary Table S11].

## Discussion

We found a 37% protective effect of carrageenan-gel use against incident HPV infections compared to placebo gel use, but no acceleration of clearance of existing HPV infections. Adherence was balanced between arms, and the study gels were generally well tolerated. These findings corroborate the interim analysis results, published in 2019, which reported a nearly identical estimate of a 36% protective effect of carrageenan against incident HPV infection (HR 0.64 [95% CI 0.45–0.89], N = 277).^9^

Clinical studies, and trials in particular, on the efficacy of carrageenan-based gels against HPV infection are scarce (Supplementary Table S12). The CATCH study represents the first phase IIB trial specifically designed to assess the efficacy of carrageenan against incident and prevalent HPV infections. Prior to its initiation, a post-hoc analysis of a trial originally designed to assess carrageenan’s anti-HIV effect found a lower prevalence of HPV at the trial’s end in compliant users of Carraguard® (carrageenan gel) compared to compliant placebo (methylcellulose) gel users (aOR 0.62 [95% CI 0.41–0.94], N = 348) adjusted for site, STI, average coital frequency, longer time in study, abnormal pap smear, baseline condom use, age by relationship, and promiscuity by condom use; however, this was a subgroup analysis, and there were no baseline nor intermediate measures of HPV to use as a reference.^19^ Other smaller trials, evaluated carrageenan’s anti-HPV activity *in vitro* from a cervicovaginal lavage using combination products of MIV-150, zinc acetate dihydrate, and carrageenan (20 participants),^20^ as well as carrageenan and griffithsin (13 participants),^21^ demonstrating anti-HPV activity in all or the majority of samples. Moreover, while there was evidence for carrageenan preventing vaginal HPV infections, carrageenan-based gels have not been shown to be effective against anal HPV infections in men,^22^ possibly due in part to anatomical differences between the vagina and anal canal, as well as histological differences: stratified squamous in the vagina and simple columnar and stratified squamous epithelium in the anal canal.

Adherence to the intervention was balanced by arm. We would expect to see that more compliant participants would have increased protection against incident HPV infections. Another trial, assessing Carraguard®’s anti-HPV activity post-hoc, found a protective effect of carrageenan against prevalent HPV infections but only among adherent users (20.3%, 348/1718).^19^ However, in the CATCH trial, a dose-response relationship was not observed. This could be due to misclassification of adherence. For example, during the first month of participation, participants were asked to apply the gel every other day for the purpose of assessing if the gel could increase clearance of existing infections. If a participant reported vaginal intercourse but no gel use during vaginal intercourse, but used the gel outside of vaginal intercourse, she would be classified as non-compliant but may have been offered some protection against HPV infection due to residual carrageenan gel in the vaginal canal. One study showed that carrageenan is present 8–24 h after Carraguard® gel application for the majority of participants (75%, 12/16), based on cervicovaginal lavage samples.^23^ In a study that tested Divine9™, carrageenan was detected in mouse vaginal washes up to 4 h after application, but not after 24 h.^24^ Another study testing Divine9, but in cervicovaginal lavage samples, detected carrageenan in the majority (78%) of samples 8 h after gel application.^25^ Based on these studies, it is reasonable to expect that there would at least be some anti-HPV activity several hours post-application, suggesting that if a woman applied the gel in the morning and had intercourse in the evening that she could have been offered some protection.

The assessment of gel safety, comparing Divine9 (intervention) to Divine (placebo), found 37% of participants reported AEs. A large trial (N = 6202), comparing Carraguard® (a carrageenan-based gel) to methylcellulose with up to 2 years of follow-up, reported that 23% of participants experienced AEs.^7^ This indicates worse tolerability of the study gels used in the CATCH trial compared to the Carraguard® trial. Over the course of follow-up, 16.5% (70/425) of CATCH participants reported a vaginal yeast infection and 5.6% (24/427) reported having bacterial vaginosis. These proportions are lower than those reported in a phase II safety trial (N = 165), comparing Carraguard® to methylcellulose, where 38% of participants reported having a yeast infection and 22% reported having bacterial vaginosis over follow-up.^26^ The difference may be explained by the nature of reporting; these conditions were self-reported in the CATCH study, whereas they were systematically tested for at each study visit in the safety trial.^26^ The nature of reporting may have led to detection bias (i.e., systematically testing for these conditions leading to more detections), as bacterial vaginosis can be asymptomatic and therefore not self-reported by participants.

While the study gels were approved by Health Canada and have received 510(k) clearance by the United Stated Food and Drug Administration (FDA), they are hyperosmolal and therefore do not meet the current WHO recommendation for osmolality (<1200 mOsM/kg).^27^ Hyperosmolality and certain ingredients, such as polyquaternium-15 and nonoxynol-9, have been shown to increase epithelial exfoliation, or sloughing, and cause cytotoxicity, potentially increasing the risk of acquiring certain STIs.^27^ However, these products remain commercially available. Studying the tolerability of lubricants and identifying the ingredients responsible for causing irritation is a challenge, as conclusive evidence on the safety and harms associated with personal lubricant is difficult to generate due to a high number of potential confounders, such as condom use, frequency of sex, and differences in reporting of lubricant use. For example, a lack of evidence of harm caused by polyquaternium led the WHO in 2020 to revert its 2012 recommendation to avoid polyquaternium in personal lubricants.^27^ The study gels were later reformulated using the same type of carrageenan at the same concentration but with adjustments to inactive ingredients to meet the WHO recommendations on osmolality. The reformulated gel was not used in the CATCH trial (Dean Fresonke, Personal communication). Use of the reformulated gel may improve adherence, and reduce difficulties in using the gel.

The trial was not without limitations. First, gel use was self-reported, which may be subject to social desirability bias if participants tended to overreport adherence to gel use. This could possibly lead to misclassification of exposure and would be expected to bias the estimate towards the null in adherence analyses. Second, the proportion of AEs experienced by participants in the carrageenan arm might have been underestimated. Comparing participants in carrageenan to placebo arm, there were fewer participants randomised (227 vs. 234), more participants who discontinued participation after their first visit (19/227 vs. 13/234), and fewer participants who reported AEs (19/227 vs. 9/234), leading to fewer participants available to report AEs in the carrageenan arm. While AEs were balanced between arms, this does not exclude the possibility that the study gels could have caused more irritation than would be expected from other common lubricants or methylcellulose, which have been used in other microbicide trials. Thirdly, the placebo gel, while identical to the intervention gel except for the addition of carrageenan, may not be a true placebo, as it could have anti-HPV activity that would bias the estimate towards the null. Fourth, the true effect of gel use may be closer to the null due to the chance that carrageenan could inhibit detectability in a small proportion of samples. Fifth, although there was considerable loss to follow-up, the median follow-up time of participants who withdrew was 3.2 months, and 40.2% (82/204) participants who withdrew experienced the outcome (not shown). Therefore, we expect any bias from loss to follow-up to be minimal. Sixth, there were imbalances between groups at baseline, however, adjusting for these imbalances and for adherence did not change the point estimate significantly. Additional limitations inherent in RCTs include measurement bias in ITT estimates, as well as selection bias introduced 1) in per-protocol estimates, 2) by the use of a hazard ratio, and 3) due to missing outcome data. We attempted to overcome the aforementioned limitations by incorporating sensitivity analyses, including best-worst/worst-best scenarios and IPW. The results of which were consistent with a protective effect.

The greatest strength of the trial is that it is the first phase IIB randomised controlled trial designed to assess the efficacy of a carrageenan-based gel on incident and prevalent HPV infections. A strength of having two identical gels, with the exception of the addition of carrageenan, is that the protective effect observed can be attributed to the presence of carrageenan. An additional strength is the collection of detailed information over time, in particular the implementation of a daily calendar to obtain details of gel use, sexual activity, and adverse events, which lead to the collection of 95,327 observations. HPV positivity was assessed by detecting 36 different HPV types using the Linear Array, which allowed us to assess genotype-specific HPV incidence among HPV-positive women.

It has been hypothesised that carrageenan works by binding directly to the HPV capsid, thereby preventing HPV from binding to heparan sulfate proteoglycan (HSPG), which is a key step in the infection process on the basement membrane.^28^ Carrageenan has also been shown to prevent attachment of HPV to human sperm,^29^ potentially preventing dispersion of HPV within the vaginal canal. Carrageenan may also act through a secondary mechanism independent of HSPG, where carrageenan prevents the virus from binding secondary receptors during the infection process.^28^^,^^30^

Multiple mechanisms may be responsible for the anti-HPV activity demonstrated in the CATCH study.

This research supports current investigations on the use and utility of carrageenan co-formulated with other anti-microbials (such as griffithsin and carrageenan) in multi-prevention technologies,^21^ as well as with MIV-150, consisting of an anti-HIV agent, zinc acetate and carrageenan.^20^ To improve adherence to the intervention and encourage STI protection in general, it may be equally important to also study use of carrageenan-based gels in pre-packaged condoms.

Despite the high osmolality of the study gels, carrageenan was protective against incident HPV infections in women. Given that carrageenan-based gels have not demonstrated efficacy in preventing anal HPV infections in men, it would be necessary to conduct a gel tolerability study with the reformulated carrageenan-based gel at the correct osmolality in both women and men that include measurements of anal HPV.

In conclusion, carrageenan compared to placebo gel use was associated with a reduction in incident HPV infections. Topical microbicides against HPV, such as carrageenan-based gels, may have utility as a complement to HPV vaccination among sexually active women.

## Contributors

The authors contributed to: conceptualisation (ELF, ANB, FC, P-PT, JET, CL, MZ); data curation (MZ, CL, SBP); formal analysis (CL); funding acquisition (ELF, ANB, FC, P-PT, JET); investigation (ELF, MZ, CL, SBP, FC), methodology (ELF, ANB, FC, P-PT, JET, MZ, CL); project administration (ELF, MZ); resources (ELF, MZ, FC); supervision (ELF, MZ); validation (CL, SBP); visualisation (CL); writing—original draft (CL); and writing—reviewing and editing (ELF, ANB, FC, P-PT, JET, MZ, SBP, CL). CL and SBP directly assessed and verified the underlying data. All authors had full access to the study data and had the final responsibility to decide to submit for publication.

## Data sharing statement

The sample code used to generate the results are available in the McGill Dataverse repository (https://doi.org/10.5683/SP3/0DS6FP). Following publication, the anonymised study data and a codebook will be made also available. The study protocol and accompanying documents, such as the informed consent form were previously published.^8^

## Declaration of interests

ELF reports grants and personal fees from Merck, grants, personal fees and non-financial support from Roche, and personal fees from GSK, outside the submitted work. ELF and MZ hold a patent related to the discovery “DNA methylation markers for early detection of cervical cancer”, registered at the Office of Innovation and Partnerships, McGill University, Montreal, Quebec, Canada (October 2018). FC reports grants from Réseau FRQS-SIDA during the conduct of the study and grants to his institution for HPV-related work from Merck Sharp and Dome, Roche Diagnostics and Becton Dickinson, outside of the submitted work. JET is an employee of Merck Sharp & Dohme LLC, a subsidiary of Merck & Co., Inc, Rahway, NJ, USA. P-PT, ANB, SBP, and CL have nothing relevant to this article to declare.
