# Supplementary material for: Efficacy and safety of a self-applied carrageenan-based gel to prevent human papillomavirus infection in sexually active young women (CATCH study): an exploratory phase IIB randomised, placebo-controlled trial
Source: eClinicalMedicine. 2023 Jun 8;60:102038. doi: 10.1016/j.eclinm.2023.102038 (PMC10314142; doi:10.1016/j.eclinm.2023.102038)
Supplement: Supplementary Figs. S1–S4 and Tables S1–S12 [file mmc1.pdf]

Supplementary Table 1. Summary of differences between protocol and current manuscript

Discrepancy	What was written in the grant protocol	What was written in the published protocol[a]	Current manuscript
description of randomization was inconsistent	randomly variable block sizes	block-size of 8	block randomisation with randomly variable block sizes (up to a block size of 8)
description of models was not pre-specified	no specification of using clustered or stratified Cox models	no specification of using clustered or stratified Cox models	used a model clustered by participant and stratified by HPV type for type-specific analyses
source of adverse events was not pre-specified	source not specified	source not specified	adverse event information was tabulated from the daily calendar, follow-up surveys, adverse event module, and adverse event follow-up
change in adherence definition	adherent if participant reported gel use as recommended in >50% of all intercourse acts	adherent if participant reported gel use as recommended in >50% of all intercourse acts	cumulative adherence >50% prior to failure/censoring[b]
description of trial completion was inconsistent	participant will be considered to have completed the study after completing 12 months of follow-up, and after her final set of data has been collected and entered	not specified	participants were considered to have completed the study if they attended all 7 study visits (irrespective of duration)
change in description of primary and secondary outcomes	primary outcome 2 is clearance of infections with HPV types observed at baseline	primary outcome 2 is HPV type-specific clearance of infection(s) detected at enrollment	the secondary outcome was HPV type-specific clearance of infection(s) detected at enrollment[c]

[a] Protocol paper was previously published: Laurie et al. 2021 *Contemp Clin Trials*.

[b] Due to presence of missing data and difficulty categorizing participants who did not report sex in the study interval, cumulative compliance to gel use was calculated in place of overall adherence to minimize missing data.

[c] Due to the original sample size calculation and analysis plan, it was more appropriate to define clearance as a secondary outcome.

Data collection and results using the daily calendar

The purpose of the CATCH calendar was to track participants' daily sexual activity, condom use, and compliance to the intervention. It was designed to be user friendly while conferring some degree of privacy through the use of picture icons (**Figure 1**). The calendar interface was set up to allow participants, when logging in securely through a computer or mobile device, to update their information. To minimize recall bias, participants were asked to update information for any given day within seven days, after which they were unable to view or modify that day's information. Thus, at any time, participants were able to enter or modify information for the seven previous days, i.e. participants needed to log in at least once a week.

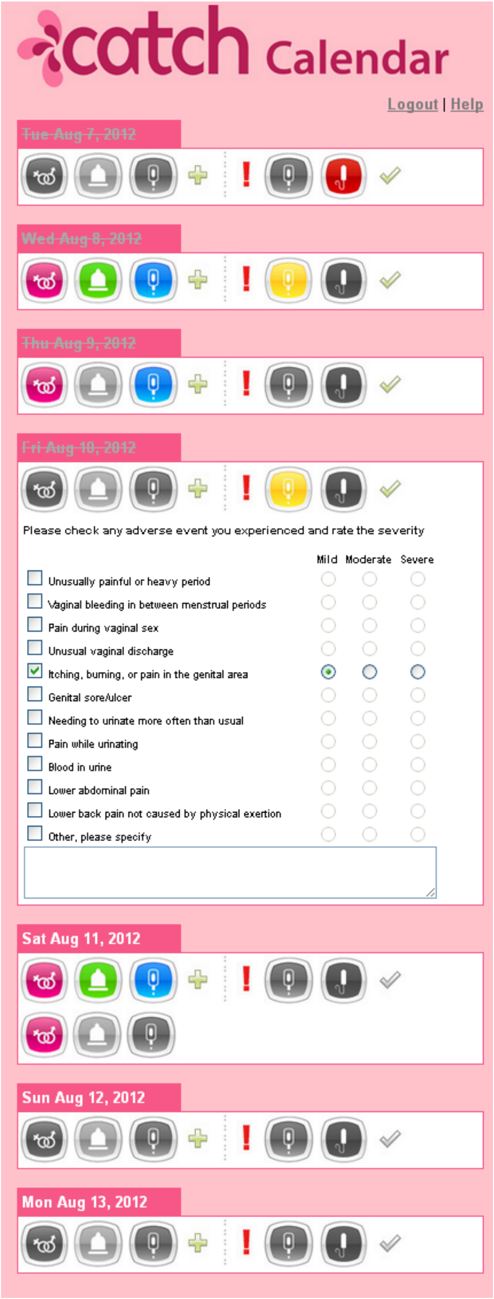

Supplementary Figure 1. Interface of the CATCH daily calendar

<u>Supplementary Figure 1 Legend</u>

Figure 1 shows an example of the daily calendar. Each icon has a mouse-over description. Using the top row as an example , the icons in sequence read:

"Press to indicate vaginal intercourse with a male partner on this day"

"Press if condoms were used during vaginal intercourse"

"Press if the CATCH gel was used before or during sexual activity"

"Press if you engaged in vaginal intercourse more than once in this day"

"Press to report adverse reactions to gel use"

"Press if the CATCH gel was used vaginally while not engaging in sexual activity" "Press if you were menstruating on this day"

"Press to cross out days that have passed" or if the. day is already crossed out "Press to edit this day's information".

The icons change from grey to colored when they are pressed. Strikethrough indicates the date is in the past.

If the participant pressed an icon to report adverse events (see example for August 10, 2012), the participant could then click a box to indicate which adverse event(s) they experienced, and also specify whether the adverse event was mild, moderate, or severe.

Supplementary Table 2A. Number of entries in the daily calendar, by study arm. The number of entries corresponds to the number of days participants filled in the calendar. The total number of follow-up days was 147,888.

	Carrageenan	Placebo
Number of entries	46,902	48,425

Supplementary Table 2B. Adherence to daily calendar use, by study arm. Adherence was calculated as the number of entries made between two consecutive study visits divided by the actual number of days between a study interval, multiplied by 100.

Adherence to calendar use, n (%)	Carrageenan (Number of follow-up study visits=988)	Placebo (Number of follow-up study visits=1068)
<25	64 (6·5)	73 (6·8)
25 – 49·9	81 (8·2)	91 (8·5)
50 – 74·9	148 (15·0)	164 (15·4)
75 – 99.9	418 (42·3)	483 (45·2)
100	202 (20·5)	172 (16·1)
No entries in interval	75 (7·6)	85 (8·0)

Supplementary Table 2C. Adherence to gel use, by study arm. Adherence was calculated as the number of gel usages before/after vaginal intercourse divided by the number of vaginal intercourses within the study interval, multiplied by 100.

Adherence to gel use, n (%)	Carrageenan (Number of follow-up study visits=988)	Placebo (Number of follow-up study visits=1068)
0	141 (14·3)	165 (15·5)
>0 – 24.9	43 (4·4)	57 (5·3)
25 – 49·9	88 (8·9)	83 (7·8)
50 – 74·9	112 (11·3)	145 (13·6)
75 – 99.9	103 (10·4)	90 (8·4)
100	187 (18·9)	214 (20·0)
No intercourse in interval	242 (24·5)	231 (21·6)
No entries in interval	72 (7·3)	83 (7·8)

Validation of HPV vaccination status

At the time of interim analysis, participants were emailed to confirm their HPV vaccination status. However, this procedure was later discontinued – starting September 25, 2019 – as the baseline and follow-up surveys were amended to include more detailed questions about HPV vaccination status, including the number of doses, brand name of the vaccine, and date of the first vaccination dose. There could be detection bias, as we did not email participants who reported not being vaccinated to verify their HPV vaccination status.

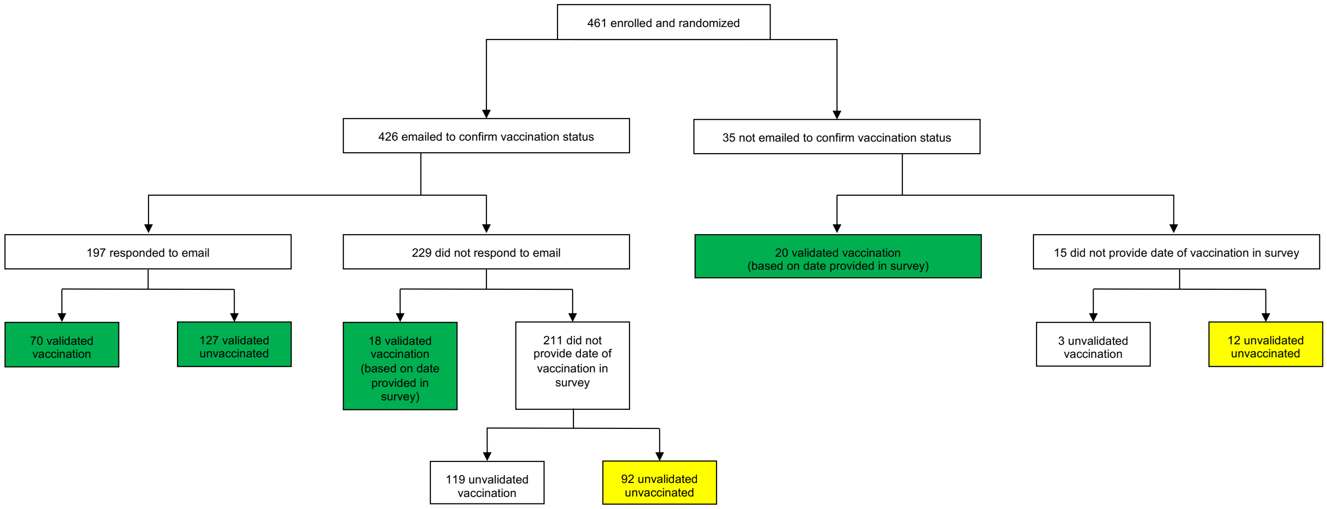

Supplementary Figure 2. Flowchart of participants through different methods of verifying HPV vaccination status

Supplementary Figure 2 Legend

Of the 426 participants emailed to confirm their vaccination status, 46·2% (197/426) responded to the email. Of the 229 who did not respond, 18 had provided the date of vaccination in the baseline or follow-up survey. Of the 35 participants who were not emailed, 57·1% (20/35) provided their date of vaccination in the baseline or follow-up survey. Boxes in green correspond to the most conservative validated definition of HPV vaccination status; these were included in the main Figure 1 and Table 3 of the manuscript. The combination of green and yellow boxes represent the liberal definition of HPV vaccination status.

Supplementary Table 3A. HPV vaccination status reported in baseline survey or at screening, no validation

Vaccination status at baseline, n (%)	Carrageenan (n=227)	Placebo (n=234)
Vaccinated	97 (42·7)	120 (51·3)
Unvaccinated	130 (57·3)	114 (48·7)

Imbalance but not significant (p = 0.066, χ^2 test).

Supplementary Table 3B. Comparison of HPV vaccination status at baseline to the validated vaccination status for participants who were emailed

	Validated status	
Vaccination status at baseline, n (%)	Vaccinated (n=70)	Unvaccinated (n=127)
Vaccinated	56	4
Unvaccinated	14	123

We changed the HPV vaccination for 14 participants from unvaccinated to vaccinated and for 4 participants from vaccinated to unvaccinated.

Supplementary Table 3C. Comparison of HPV vaccination using different definitions of validation from different sources

Validation description	HPV vaccination status at baseline	Carrageenan	Placebo
Validated by email		n=97	n=100
	Vaccinated	33 (34·0)	37 (37·0)
	Unvaccinated	64 (66·0)	63 (63·0)
Validated by email or by providing dates of vaccination in survey(s)		n=111	n=124
	Vaccinated	47 (42·3)	61 (49·2)
	Unvaccinated	64 (57·7)	63 (50·8)
Validated by email, by providing dates of vaccination in survey(s), or reporting they were unvaccinated in survey(s)		n=174	n=165
	Vaccinated	47 (27·0)	61 (37·0)
	Unvaccinated	127 (73·0)	104 (63·0)

Supplementary Figure 3. Distribution of time between baseline and subsequent visits compared to the study schedule, by study arm

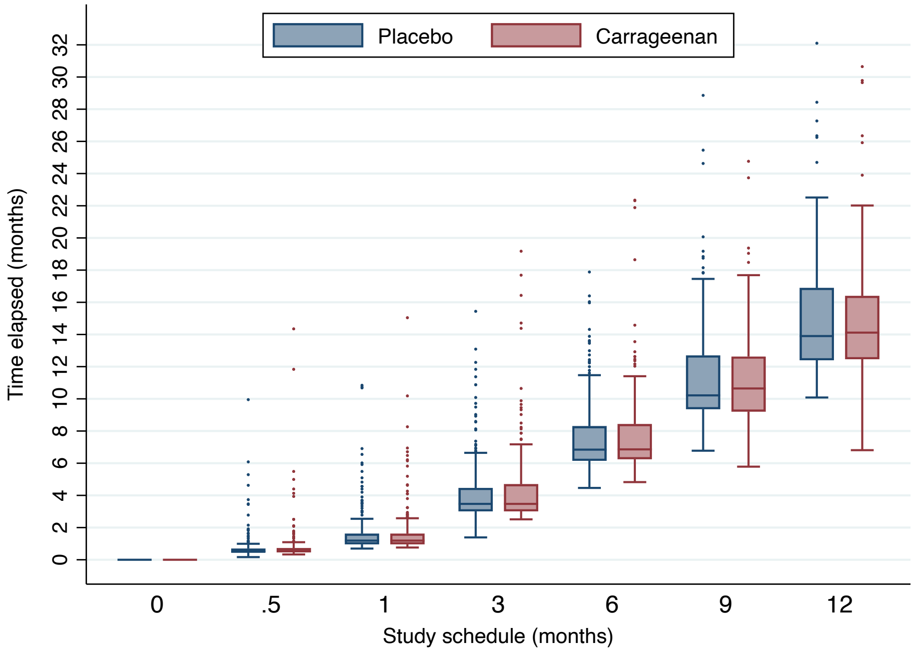

<u>**Supplementary Figure 3 Legend**</u>

The box plot shows on the x-axis the planned study schedule (baseline study visit and 6 follow-up visits at 0.5, 1, 3, 6, 9 and 12 months) relative to the actual time that elapsed between study visits on the y-axis. The midline of each box represents the median, the ends of the box represent the first and third quartile, and the lower and upper whiskers range from the minimum to the maximum values observed (excluding outliers). The individual dots represent outliers: values that are either 1.5 box-lengths (i.e., inter-quartile ranges) above or below the box.

Supplementary Figure 4. Sub-group analyses of incidence/detection of HPV infections

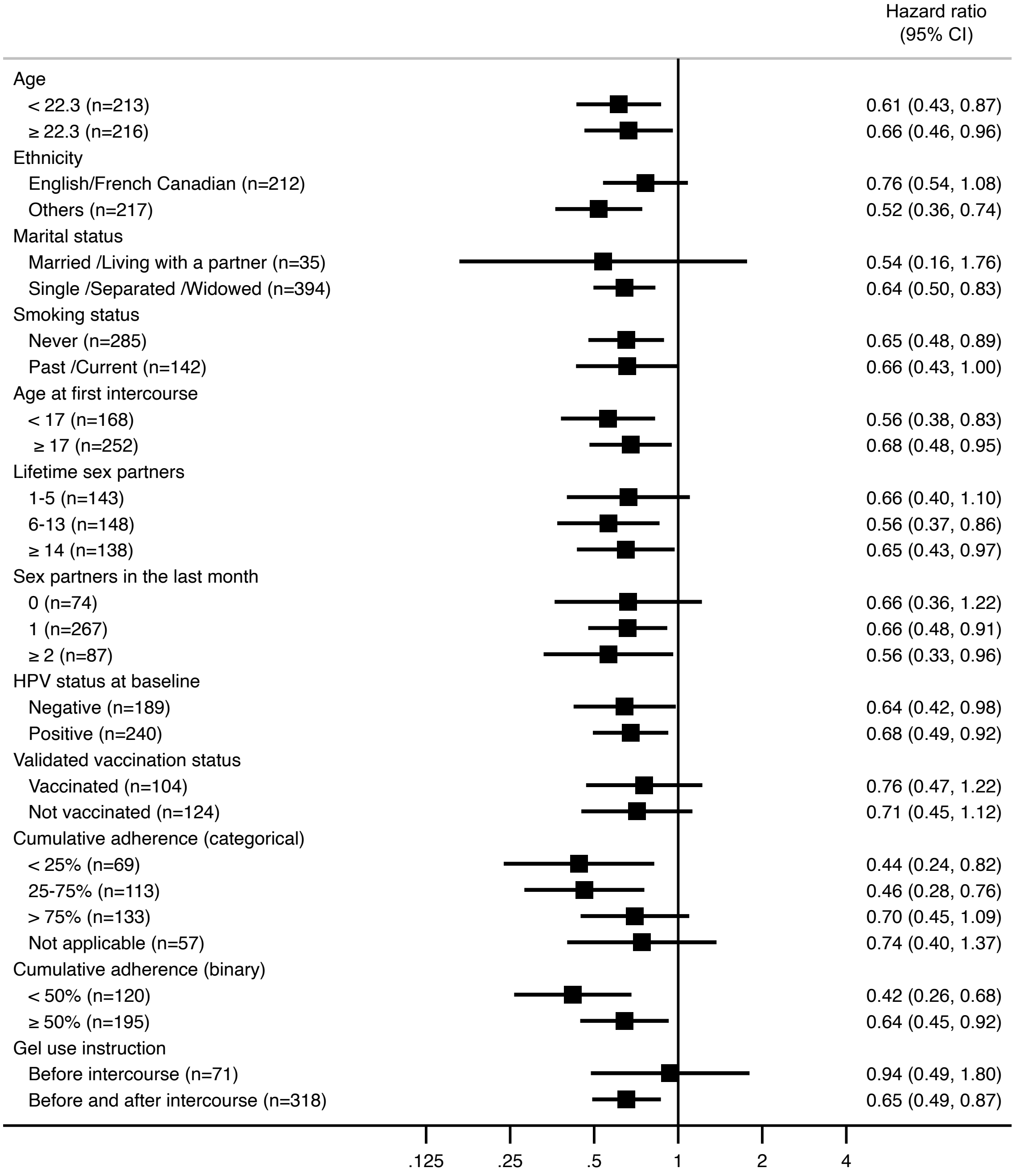

<u>**Supplementary Figure 4 Legend**</u>

The forest plot shows the results from sub-group analyses for the purpose of comparing the effect of carrageenan according to main baseline characteristics, gel instruction at the time of enrollment, and cumulative adherence to follow-up based on calendar data. The cut-off for age and age at first intercourse was based on the respective median value of these variables.

Supplementary Table 4A. HPV prevalence [n, (%)] by visit number in carrageenan arm

HPV type/groups	Visit 1 (n=227)	Visit 2 (n=208)	Visit 3 (n=190)	Visit 4 (n=171)	Visit 5 (n=152)	Visit 6 (n=140)	Visit 7 (n=127)
**HPV6**	5 (2·2)	4 (2·0)	5 (2·7)	5 (2·9)	3 (2·0)	3 (2·1)	2 (1·6)
**HPV11**	1 (0·5)	1 (0·5)	1 (0·5)	2 (1·2)	1 (0·7)	0 (0)	0 (0)
**HPV16**	8 (3·6)	11 (5·5)	10 (5·4)	12 (7·1)	10 (6·6)	8 (5·7)	6 (4·8)
**HPV18**	3 (1·3)	2 (1·0)	2 (1·1)	1 (0·6)	2 (1·3)	1 (0·7)	2 (1·6)
**HPV26**	0 (0)	0 (0)	0 (0)	1 (0·6)	1 (0·7)	1 (0·7)	2 (1·6)
**HPV31**	6 (2·7)	3 (1·5)	5 (2·7)	6 (3·5)	4 (2·7)	4 (2·9)	4 (3·2)
**HPV33**	1 (0·5)	0 (0)	1 (0·5)	1 (0·6)	2 (1·3)	3 (2·1)	0 (0)
**HPV34**	0 (0)	0 (0)	0 (0)	0 (0)	0 (0)	0 (0)	0 (0)
**HPV35**	4 (1·8)	0 (0)	0 (0)	1 (0·6)	2 (1·3)	1 (0·7)	2 (1·6)
**HPV39**	13 (5·8)	10 (5·0)	12 (6·4)	13 (7·7)	12 (8·0)	10 (7·1)	12 (9·5)
**HPV40**	7 (3·1)	6 (3·0)	4 (2·1)	3 (1·8)	3 (2·0)	5 (3·6)	4 (3·2)
**HPV42**	17 (7·6)	13 (6·4)	10 (5·4)	12 (7·1)	10 (6·6)	9 (6·4)	9 (7·1)
**HPV44**	6 (2·7)	4 (2·0)	3 (1·6)	3 (1·8)	3 (2·0)	3 (3·6)	2 (1·6)
**HPV45**	6 (2·7)	3 (1·5)	2 (1·1)	4 (2·4)	3 (2·0)	5 (3·6)	3 (2·4)
**HPV51**	22 (9·8)	16 (7·9)	13 (7·0)	12 (7·1)	8 (5·3)	10 (7·1)	8 (6·4)
**HPV52**	17 (7·6)	12 (5·9)	11 (5·9)	10 (5·9)	9 (6·0)	7 (5·0)	6 (4·8)
**HPV53**	22 (9·8)	18 (8·9)	15 (8·0)	13 (7·7)	12 (8·0)	12 (8·6)	8 (6·4)
**HPV54**	10 (4·5)	8 (4·0)	6 (3·2)	9 (5·3)	15 (9·9)	10 (7·1)	9 (7·1)
**HPV56**	15 (6·7)	16 (7·9)	13 (7·0)	10 (5·9)	6 (4·0)	6 (4·3)	4 (3·2)
**HPV58**	12 (5·4)	9 (4·5)	8 (4·3)	6 (3·5)	7 (4·6)	4 (2·9)	4 (3·2)
**HPV59**	11 (4·9)	12 (5·9)	11 (5·9)	10 (5·9)	10 (6·6)	11 (7·9)	8 (6·4)
**HPV61**	9 (4·0)	7 (3·5)	8 (4·3)	8 (4·7)	6 (4·0)	4 (2·9)	5 (4·0)
**HPV62**	14 (6·3)	11 (5·5)	9 (4·8)	6 (3·5)	7 (4·6)	5 (3·6)	8 (6·4)
**HPV66**	20 (8·9)	17 (8·4)	12 (6·4)	12 (7·1)	13 (8·6)	8 (5·7)	7 (5·6)
**HPV67**	6 (2·7)	6 (3·0)	6 (3·2)	11 (6·5)	6 (4·0)	4 (2·9)	4 (3·2)
**HPV68**	5 (2·2)	5 (2·5)	5 (2·7)	3 (1·8)	1 (0·7)	1 (0·7)	3 (2·4)
**HPV69**	1 (0·5)	2 (1·0)	2 (1·1)	1 (0·6)	0 (0)	1 (0·7)	0 (0)
**HPV70**	3 (1·3)	4 (2·0)	2 (1·1)	0 (0)	0 (0)	0 (0)	0 (0)
**HPV71**	0 (0)	0 (0)	0 (0)	0 (0)	0 (0)	1 (0·7)	1 (0·8)
**HPV72**	1 (0·5)	1 (0·5)	0 (0)	0 (0)	1 (0·7)	1 (0·7)	1 (0·8)
**HPV73**	5 (2·2)	7 (3·5)	5 (2·7)	7 (4·1)	9 (6·0)	4 (2·9)	5 (4·0)
**HPV81**	6 (2·7)	2 (1·0)	5 (2·7)	3 (1·8)	3 (2·0)	3 (2·1)	4 (3·2)
**HPV82**	4 (1·8)	0 (0)	2 (1·1)	2 (1·2)	3 (2·0)	6 (4·3)	5 (4·0)
**HPV83**	3 (1·3)	3 (1·5)	3 (1·6)	4 (2·4)	6 (4·0)	5 (3·6)	3 (2·4)
**HPV84**	20 (8·9)	21 (10·4)	19 (10·2)	20 (11·8)	13 (8·6)	13 (9·3)	6 (4·8)
**HPV89**	22 (9·8)	22 (10·9)	17 (9·1)	19 (11·2)	14 (9·3)	9 (6·4)	9 (7·1)
**Negative**	109 (48·7)	100 (49·5)	92 (49·2)	76 (44·7)	70 (46·4)	69 (49·3)	61 (48·4)
**Any HPV**	**115 (50·5)**	**102 (50·5)**	**95 (50·8)**	**94 (55·3)**	**81 (53·6)**	**71 (50·7)**	**65 (51·6)**
**Subgenus 1[a]**	38 (17·0)	31 (15·4)	27 (14·4)	30 (17·7)	30 (19·9)	26 (18·6)	19 (15·1)
**Subgenus 2[b]**	96 (42·9)	81 (40·1)	75 (40·1)	73 (42·9)	61 (40·4)	60 (42·9)	53 (42·1)
**Subgenus 3[c]**	60 (26·8)	52 (25·7)	46 (24·6)	47 (27·7)	37 (24·5)	30 (21·4)	31 (24·6)
**Missing[d]**	3 (1·3)	6 (2·9)	3 (1·6)	1 (0·6)	1 (0·7)	0 (0)	1 (0·8)

[a] Subgenus 1 group consists of HPVs 6, 11, 40, 42, 44, and 54.

[b] Subgenus 2 group consists of HPVs 16, 18, 26, 31, 33, 34, 35, 39, 45, 51, 52, 53, 56, 58, 59, 66, 67, 68, 69, 70, 73, and 82.

[c] Subgenus 3 group consists of HPVs 61, 62, 71, 72, 81, 83, 84, and 89.

[d] Missing results correspond to invalid or mishandled samples. Missing samples were not included in percentages.

Supplementary Table 4B. HPV prevalence [n, (%)] by visit number in placebo arm

HPV type/groups	Visit 1 (n=234)	Visit 2 (n=221)	Visit 3 (n=209)	Visit 4 (n=191)	Visit 5 (n=166)	Visit 6 (n=151)	Visit 7 (n=130)
**HPV6**	5 (2·1)	5 (2·3)	5 (2·4)	5 (2·6)	6 (3·7)	6 (4·0)	5 (3·9)
**HPV11**	0 (0)	0 (0)	0 (0)	1 (0·5)	1 (0·6)	1 (0·7)	1 (0·8)
**HPV16**	10 (4·3)	12 (5·4)	12 (5·8)	10 (5·3)	11 (6·7)	10 (6·6)	7 (5·4)
**HPV18**	6 (2·6)	3 (1·4)	4 (1·9)	4 (2·1)	2 (1·2)	4 (2·7)	2 (1·5)
**HPV26**	1 (0·4)	1 (0·5)	0 (0)	1 (0·5)	1 (0·6)	0 (0)	0 (0)
**HPV31**	7 (3·0)	6 (2·7)	10 (4·8)	11 (5·8)	9 (5·5)	10 (6·6)	1 (0·8)
**HPV33**	2 (0·9)	4 (1·8)	3 (1·4)	4 (2·1)	3 (1·8)	1 (0·7)	0 (0)
**HPV34**	1 (0·4)	1 (0·5)	1 (0·5)	1 (0·5)	0 (0)	1 (0·7)	3 (2·3)
**HPV35**	7 (3·0)	6 (2·7)	6 (2·9)	4 (2·1)	3 (1·8)	3 (2·0)	3 (2·3)
**HPV39**	16 (6·8)	15 (6·8)	16 (7·7)	9 (4·7)	12 (7·3)	11 (7·3)	10 (7·7)
**HPV40**	8 (3·4)	7 (3·2)	10 (4·8)	11 (5·8)	6 (3·7)	7 (4·6)	3 (2·3)
**HPV42**	23 (9·8)	26 (11·8)	20 (9·6)	17 (9·0)	16 (9·8)	18 (11·9)	17 (13·1)
**HPV44**	8 (3·4)	13 (5·9)	10 (4·8)	11 (5·8)	5 (3·1)	7 (4·6)	3 (2·3)
**HPV45**	6 (2·6)	5 (2·3)	5 (2·4)	6 (3·2)	7 (4·3)	6 (4·0)	2 (1·5)
**HPV51**	34 (14·5)	30 (13·6)	25 (12·0)	23 (12·1)	16 (9·8)	14 (9·3)	8 (6·2)
**HPV52**	13 (5·6)	16 (7·2)	11 (5·3)	11 (5·8)	9 (5·5)	9 (6·0)	11 (8·5)
**HPV53**	31 (13·3)	32 (14·5)	25 (12·0)	28 (14·7)	28 (17·1)	18 (11·9)	15 (11·5)
**HPV54**	18 (7·7)	15 (6·8)	16 (7·7)	18 (9·5)	16 (9·8)	12 (8·0)	14 (10·8)
**HPV56**	6 (2·6)	6 (2·7)	6 (2·9)	7 (3·7)	3 (1·8)	6 (4·0)	3 (2·3)
**HPV58**	13 (5·6)	13 (5·9)	10 (4·8)	10 (5·3)	12 (7·3)	11 (7·3)	9 (6·9)
**HPV59**	19 (8·1)	18 (8·1)	15 (7·2)	12 (6·3)	14 (8·5)	8 (5·3)	5 (3·9)
**HPV61**	10 (4·3)	10 (4·5)	12 (5·8)	15 (7·9)	12 (7·3)	9 (6·0)	6 (4·6)
**HPV62**	21 (9·0)	19 (8·6)	12 (5·8)	21 (11·1)	18 (11·0)	15 (9·9)	13 (10·0)
**HPV66**	23 (9·8)	27 (12·2)	22 (10·6)	18 (9·5)	11 (6·7)	12 (8·0)	12 (9·2)
**HPV67**	13 (5·6)	13 (5·9)	15 (7·2)	11 (5·8)	8 (4·9)	4 (2·7)	5 (3·9)
**HPV68**	6 (2·6)	7 (3·2)	4 (1·9)	3 (1·6)	4 (2·4)	3 (2·0)	2 (1·5)
**HPV69**	0 (0)	0 (0)	0 (0)	0 (0)	0 (0)	0 (0)	1 (0·8)
**HPV70**	3 (1·3)	4 (1·8)	5 (2·4)	2 (1·1)	3 (1·8)	3 (2·0)	1 (0·8)
**HPV71**	0 (0)	0 (0)	0 (0)	0 (0)	0 (0)	1 (0·7)	1 (0·8)
**HPV72**	0 (0)	1 (0·5)	0 (0)	0 (0)	0 (0)	0 (0)	1 (0·8)
**HPV73**	14 (6·0)	14 (6·3)	12 (5·8)	9 (4·7)	10 (6·1)	12 (8·0)	6 (4·6)
**HPV81**	8 (3·4)	7 (3·2)	6 (2·9)	6 (3·2)	5 (3·1)	2 (1·3)	6 (4·6)
**HPV82**	7 (3·0)	6 (2·7)	11 (5·3)	9 (4·7)	7 (4·3)	8 (5·3)	1 (0·8)
**HPV83**	6 (2·6)	5 (2·3)	6 (2·9)	5 (2·6)	5 (3·1)	5 (3·3)	6 (4·6)
**HPV84**	29 (12·4)	25 (11·3)	25 (12·0)	21 (11·1)	18 (11·0)	17 (11·3)	14 (10·8)
**HPV89**	27 (11·5)	37 (16·7)	33 (15·9)	21 (11·1)	23 (1·40)	19 (12·6)	11 (8·5)
**Negative**	93 (39·7)	78 (35·3)	73 (35·1)	64 (33·7)	57 (34·8)	60 (39·7)	56 (43·1)
**Any HPV**	**141 (60·3)**	**143 (64·7)**	**135 (64·9)**	**126 (66·3)**	**107 (65·2)**	**91 (60·3)**	**74 (56·9)**
**Subgenus 1[a]**	55 (23·5)	55 (24·9)	52 (25·0)	49 (25·8)	44 (26·8)	40 (26·8)	33 (25·4)
**Subgenus 2[b]**	114 (48·7)	117 (52·9)	110 (52·9)	103 (54·2)	89 (54·3)	74 (49·0)	55 (42·3)
**Subgenus 3[c]**	76 (32·5)	78 (35·3)	71 (34·1)	63 (33·2)	58 (35·4)	52 (34·4)	46 (35·4)
**Missing[d]**	0 (0)	0 (0)	1 (0·5)	1 (0·5)	2 (1·2)	0 (0)	0 (0)

[a] Subgenus 1 group consists of HPVs 6, 11, 40, 42, 44, and 54.
[b] Subgenus 2 group consists of HPVs 16, 18, 26, 31, 33, 34, 35, 39, 45, 51, 52, 53, 56, 58, 59, 66, 67, 68, 69, 70, 73, and 82.
[c] Subgenus 3 group consists of HPVs 61, 62, 71, 72, 81, 83, 84, and 89.
[d] Missing results correspond to invalid or mishandled samples. Missing samples were not included in percentages.

Supplementary Table 5. Characteristics of participants at follow-up, overall and by study arm

	Overall (n=461)	Carrageenan (n=227)	Placebo (n=234)
**Follow-up time, months**			
Mean (SD)	10·6 (6·8)	10·4 (7·0)	10·8 (6·6)
**Number of visits/participant**			
Mean (SD)	5·5 (2·1)	5·4 (2·2)	5·6 (1·9)
**Total number of visits/participant**			
1 visit only	32 (6·9)	19 (8·4)	13 (5·6)
2 visits only	30 (6·5)	18 (7·9)	12 (5·1)
3 visits only	37 (8·0)	19 (8·4)	18 (7·7)
4 visits only	44 (9·5)	19 (8·4)	25 (10·7)
5 visits only	27 (5·9)	12 (5·3)	15 (6·4)
6 visits only	34 (7·4)	13 (5·7)	21 (9·0)
7 visits	257 (55·8)	127 (56·0)	130 (55·6)
**Mean time between visits (SD), weeks**			
Between visit 1 and 2	3·6 (5·2)	3·8 (6·1)	3·4 (4·1)
Between visit 2 and 3	4·1 (5·0)	4·1 (4·9)	4·1 (5·0)
Between visit 3 and 4	11·6 (7·5)	11·9 (7·9)	11·4 (7·2)
Between visit 4 and 5	15·2 (6·5)	15·3 (6·4)	15·2 (6·6)
Between visit 5 and 6	16·0 (8·2)	15·9 (7·3)	16·1 (9·1)
Between visit 6 and 7	16·5 (8·7)	16·6 (8·6)	16·4 (8·7)
**Ever anal intercourse over follow-up**[a]	**(n=428)**	**(n=207)**	**(n=221)**
Yes	124 (29·0)	54 (26·1)	70 (31·7)
No	304 (71·0)	153 (73·9)	151 (68·3)
**Adherence**[b] **between consecutive visits, n (%)**	**n=2056**	**n=988**	**n=1068**
>75%	554 (27·0)	272 (27·5)	282 (26·4)
25-75%	468 (22·8)	218 (22·1)	250 (23·4)
<25%	406 (19·8)	184 (18·6)	222 (20·8)
Did not have intercourse	473 (23·0)	242 (24·5)	231 (21·6)
Not reported	155 (7·5)	72 (7·3)	83 (7·8)
**Average adherence**[c] **over all study visits, n (%)**	**n=429**	**n=208**	**n=221**
>50%	214 (49·9)	104 (50·0)	110 (49·8)
≤50%	169 (39·4)	79 (38·0)	90 (40·7)
Never reported sex during the study	38 (8·9)	21 (10·1)	17 (7·7)
Never reported	8 (1·9)	4 (1·9)	4 (1·8)

[a] Data not available for 32 participants (19 in the carrageenan and 13 in the placebo arm) due to no follow-up visits, and for 1 participant due to non-response.
[b] Adherence was defined as the number of times the gel was used before or during vaginal intercourse divided by the number of vaginal intercourses in the 7 days prior to the study visit. Observations where participants did not provide data on gel use and/or intercourse were classified as not reported. This data was obtained from the calendar. Based on the $\chi 2$ test, p=0·43.
[c] Average adherence was calculated by 1) dividing the number of gel uses by the number of intercourses in the time period between consecutive visits, then 2) adding these values across all visits and dividing by the number of study visits. Based on the $\chi 2$ test, p=0·822.
SD: standard deviation.

Supplementary Table 6. Estimated Kaplan-Meier survivor function from incidence analysis

Time, months	Number at risk		Number failed (had incident infection)		Number censored	
**Group**	**CG**	**PB**	**CG**	**PB**	**CG**	**PB**
0·3966[a]	208	221	14	27	2	2
0·5	192	192	20	33	8	6
1	164	153	20	30	13	9
3	131	114	22	27	11	13
6	98	74	12	12	7	2
9	79	60	8	7	8	7
12	63	46	8	8	27	24
15	28	14	1	1	17	7
18	10	6	1	1	3	2
21	6	3	2	1	3	0
24	1	2	0	0	0	1
27	1	1	0	0	1	1
30	0	0	0	0	0	0

CG: carrageenan, PB: placebo

[a] Month 0·3966 was chosen, as the first failure occurs on day 12.

Supplementary Table 7. Post-hoc sensitivity analyses for incidence based on complete case, best-worst/worst-best scenarios, adjustment for baseline imbalance, and using inverse probability weighting

	**Hazard Ratio (95% CI)**
Complete case[a]	0·64 (0·49-0·82)
Best-worst scenario[b]	0·62 (0·49-0·80)
Worst-best scenario[c]	0·71 (0·55-0·91)
Imbalance adjusted model[d]	0·66 (0·51-0·85)
Weighted by IPW model[e]	0·61 (0·47-0·79)

CI: confidence interval, IPW: inverse probability weighting.

[a] For the complete case analysis, only participants with valid HPV results at each study visit were included.
[b] In the best-worst scenario, participants with missing data at baseline were considered HPV-negative. Of participants with missing follow-up data, participants in the carrageenan arm were assumed to not have an incident HPV infection and participants in the placebo arm were assumed to have acquired an incident HPV infection.
[c] In the worst-best scenario, participants with missing data at baseline were considered HPV-negative. Of participants with missing follow-up data, participants in the placebo arm were assumed not have an incident HPV infection and participants in the carrageenan arm were assumed to have acquired an incident HPV infection.
[d] The model was adjusted for baseline variables (HPV prevalence, age at first sexual intercourse [binary based on median as cut-off], partners in the last month [categorical 0, 1, or multiple partners]) and the follow-up variable for adherence in the past 7 days.
[e] The model was weighted using the inverse probability weighting approach. Logistic regression was used to predict the probability of being a complete case (defined as participants with at least 1 follow-up visit with valid HPV results and with no missing baseline covariates), and included the following baseline variables, group assignment, age, ethnicity, marital status, smoking status, age at first intercourse, lifetime sex partners, sex partners in the last month, and HPV prevalence. We calculated the inverse of the probability for being a complete case, and applied these weights in a Cox regression model.

Supplementary Table 8. Adverse events reported in the calendar, overall and by grade, shown as number of adverse events reported (number of participants affected)

	Mild	Moderate	Severe	Not Graded	Total
**Carrageenan group**					
Any	89 (21)	40 (19)	24 (9)	75 (22)	228 (37)
Unusually heavy or painful period	2 (1)	1 (1)	0 (0)	0 (0)	3 (1)
Vaginal bleeding in between menstrual periods	16 (5)	0 (0)	0 (0)	1 (1)	17 (6)
Pain during vaginal sex	8 (6)	5 (4)	4 (3)	5 (3)	22 (13)
Unusual vaginal discharge	16 (5)	6 (6)	2 (2)	8 (2)	32 (11)
Itching, burning, or pain in the genital area	35 (16)	18 (9)	14 (8)	11 (5)	78 (23)
Genital sore/ulcer	1 (1)	1 (1)	0 (0)	0 (0)	2 (2)
Needing to urinate more often than usual	2 (2)	2 (2)	0 (0)	0 (0)	4 (3)
Pain while urinating	3 (3)	0 (0)	2 (2)	5 (1)	10 (6)
Blood in urine	1 (1)	0 (0)	1 (1)	3 (1)	5 (3)
Lower abdominal pain	5 (4)	5 (3)	1 (1)	0 (0)	11 (5)
Lower back pain not caused by physical exertion	0 (0)	0 (0)	0 (0)	0 (0)	0 (0)
Other	0 (0)	2 (2)	0 (0)	42 (20)	44 (21)
**Placebo group**					
Any	48 (21)	57 (22)	27 (12)	56 (23)	188 (39)
Unusually heavy or painful period	4 (4)	9 (3)	1 (1)	1 (1)	15 (8)
Vaginal bleeding in between menstrual periods	9 (5)	2 (2)	1 (1)	1 (1)	13 (8)
Pain during vaginal sex	0 (0)	3 (3)	6 (3)	1 (1)	10 (6)
Unusual vaginal discharge	9 (2)	9 (4)	3 (2)	0 (0)	21 (4)
Itching, burning, or pain in the genital area	17 (11)	19 (13)	12 (6)	5 (5)	53 (19)
Genital sore/ulcer	3 (3)	4 (1)	0 (0)	0 (0)	7 (3)
Needing to urinate more often than usual	2 (2)	2 (2)	1 (1)	5 (3)	10 (7)
Pain while urinating	0 (0)	2 (2)	1 (1)	3 (1)	6 (3)
Blood in urine	0 (0)	0 (0)	0 (0)	1 (1)	1 (1)
Lower abdominal pain	3 (3)	2 (2)	0 (0)	2 (2)	7 (3)
Lower back pain not caused by physical exertion	0 (0)	2 (1)	0 (0)	1 (1)	3 (2)
Other	1 (1)	3 (3)	2 (2)	36 (18)	42 (21)

Supplementary Table 9. Participant withdrawal overall and stratified by reporting of adverse events and reporting of difficulties with gel use, by study arm

	**Total (n=461)**	**Carrageenan (n=227)**	**Placebo (n=234)**
Overall			
Completed	257 (55·7)	127 (55·9)	130 (55·6)
Withdrew	204 (44·3)	100 (44·1)	104 (44·4)
Reported adverse events			
Completed	107 (62·2)	50 (63·3)	57 (61·3)
Withdrew	65 (37·8)	29 (36·7)	36 (38·7)
Did not report adverse events			
Completed	150 (51·9)	77 (52·0)	73 (51·8)
Withdrew	139 (48·1)	71 (48·0)	68 (48·2)
Reported difficulty using gel before, during or after intercourse[a]			
Completed	209 (62·4)	101 (63·5)	108 (61·4)
Withdrew	126 (37·6)	58 (36·5)	68 (38·6)
Did not report difficulty using gel before, during or after intercourse[a]			
Completed	48 (53.9)	26 (55·3)	22 (52·4)
Withdrew	41 (46·1)	21 (44·7)	20 (47·6)

[a] Because reporting difficulty using gel was a follow-up survey question, only participants with a follow-up visit were included in the denominator: excluded 32 participants (19 in the carrageenan and 13 in the placebo arm). An additional 5 participants (2 in the carrageenan and 3 in the placebo) were excluded due to non-response.

Supplementary Table 10. Reported difficulty using the study gel before, during, or after intercourse [n (%)] at the person- and study-visit level, by study arm

	Person-level[a]		Study visit-level[b]	
	**Carrageenan (n=206)**	**Placebo (n=218)**	**Carrageenan (n=871)**	**Placebo (n=962)**
**Ever reported difficulty with gel**	159 (77·2)	176 (80·7)	NA	NA
Application of the CATCH gel is too difficult	17 (8·3)	22 (10·1)	28 (3·2)	33 (3·4)
The packaging is too inconvenient	19 (9·2)	19 (8·7)	37 (4·3)	32 (3·3)
You did not have the CATCH gel on you at the time of intercourse	98 (47·6)	117 (53·7)	231 (26·5)	282 (29·3)
You forgot to use the CATCH gel	95 (46·1)	111 (50·9)	212 (24·3)	248 (25·8)
You did not want to use lubricants	54 (26·2)	85 (39·0)	105 (12·1)	184 (19·1)
You preferred other brands to the CATCH gel	24 (11·7)	31 (14·2)	44 (5·1)	60 (6·2)
You think that the quality of the CATCH gel is poor (e.g., odour, feel, etc.)	35 (17·0)	66 (30·3)	66 (7·6)	150 (15·6)
Use of the gel caused discomfort/adverse reactions to you	22 (10·7)	27 (12·4)	24 (2·8)	35 (3·6)
Partner(s) did not want to use lubricants	36 (17·5)	54 (24·8)	67 (7·7)	93 (9·7)
Partner(s) is/are allergic to ingredients of the CATCH gel	0 (0)	1 (0·5)	0 (0)	1 (0·1)
Partner(s) preferred other brands to the CATCH gel	18 (8·7)	16 (7·3)	34 (3·9)	22 (2·3)
Partner(s) think(s) that the quality of the CATCH gel is poor (e.g., odour, feel, etc.)	22 (10·7)	31 (14·2)	37 (4·3)	54 (5·6)
Use of the gel caused discomfort/adverse reactions to your partner(s)	5 (2·4)	5 (2·3)	5 (0·6)	6 (0·6)
Other	27 (13·1)	21 (9·6)	38 (4·4)	26 (2·7)

[a] Participants excluded: 19 participants in the carrageenan arm and 13 in the placebo arm only had 1 visit, and 2 participants in the carrageenan and 3 participants in the placebo never responded to this question.

[b] There were 117 study visits in the carrageenan and 106 in the placebo did not respond.

NA: not applicable.

Supplementary Table 11. Cumulative adherence to gel use before/during intercourse and prior to failure or censoring

Source	Adherence definition		Categorization	Number of participants	Adherence (%)	Hazard ratio (95% CI)
Follow-up survey	number of gel uses / number of vaginal intercourses reported **one week prior to study visit**	per-protocol[a]	NA	191	>50	0·69 (0·47-1·02)
		supplementary[b]	categorical	65	<25	0·57 (0·30-1·07)
				103	25-75	0·52 (0·29-0·94)
				155	>75	0·72 (0·47-1·08)
				98	NA	0·64 (0·40-1·04)
				8	missing data	NR
			binary	112	<50	0·58 (0·35-0·98)
				211	≥50	0·64 (0·44-0·92)
Calendar	cumulative number of gel uses / number of vaginal intercourses **between two consecutive study visits**	per-protocol[a]	NA	183	>50	0·60 (0·41-0·89)
		supplementary[b]	categorical	69	<25	0·44 (0·24-0·82)
				113	25-75	0·46 (0·28-0·76)
				133	>75	0·70 (0·45-1·10)
				57	NA	0·74 (0·40-1·37)
				57	missing data	1·14 (0·47-2·76)
			binary	120	<50	0·42 (0·26-0·68)
				195	≥50	0·64 (0·45-0·92)

Data on adherence was collected in both the follow-up surveys and daily calendar.

[a] These analyses were restricted to participants whose cumulative compliance to gel use was >50% prior to failure/censoring.

[b] Cumulative adherence to gel use before/during intercourse prior to failure or censoring.

Not applicable if participant did not report intercourse prior to acquiring an incident infection or censoring.

Not reported, as there were only 8 participants in this category, so Cox model was not run.

NA: not applicable, NR: not reported.

Supplementary Table 12. References of studies that reported on carrageenan's anti-HPV activity

Study type	Reference
in vitro	1. Buck CB, Thompson CD, Roberts JN, et al. Carrageenan is a potent inhibitor of papillomavirus infection. PLoS Pathog 2006; **2**: e69.<br>2. Perez-Andino J, Buck CB, Ribbeck K, Pérez-Andino J, Buck CB, Ribbeck K. Adsorption of human papillomavirus 16 to live human sperm. PLoS One 2009; **4**: e5847.<br>3. Cruz L, Meyers C. Differential dependence on host cell glycosaminoglycans for infection of epithelial cells by high-risk HPV types. PLoS One 2013; **8**: e68379.<br>4. Kwak K, Jiang R, Wang JW, Jagu S, Kirnbauer R, Roden RBS. Impact of inhibitors and L2 antibodies upon the infectivity of diverse alpha and beta human papillomavirus types. PLoS One 2014; **9**: e97232.<br>5. Lal M, Lai M, Ugaonkar S, et al. Development of a Vaginal Fast-Dissolving Insert Combining Griffithsin and Carrageenan for Potential Use Against Sexually Transmitted Infections. J Pharm Sci 2018; **107**: 2601–10.<br>6. Wang S, Lu Z, Wang S, et al. The inhibitory effects and mechanisms of polymannuroguluronate sulfate against human papillomavirus infection in vitro and in vivo. Carbohydr Polym 2020; **241**: 116365.
in vitro and *ex vivo*	1. Ugaonkar SR, Wesenberg A, Wilk J, et al. A novel intravaginal ring to prevent HIV-1, HSV-2, HPV, and unintended pregnancy. J Control Release; 2015. p. 57–68.<br>2. Novetsky AP, Keller MJ, Gradissimo A, et al. In vitro inhibition of human papillomavirus following use of a carrageenan-containing vaginal gel. Gynecol Oncol 2016; **143**: 313–8.<br>3. Friedland BA, Hoesley CJ, Plagianos M, et al. First-in-Human Trial of MIV-150 and Zinc Acetate Coformulated in a Carrageenan Gel. J Acquir Immune Defic Syndr 2016; **73**: 489–96.<br>4. Teleshova N, Keller MJ, Romero JAF, et al. Results of a phase 1, randomized, placebocontrolled first-in-human trial of griffithsin formulated in a carrageenan vaginal gel. PLoS One; 2022; **17**: e0261775.
in vitro and *in vivo*	1. Rodriguez A, Kleinbeck K, Mizenina O, et al. In vitro and in vivo evaluation of two carrageenan-based formulations to prevent HPV acquisition. Antiviral Res 2014; **108**: 88–93.<br>2. Wang JW, Jagu S, Kwak K, et al. Preparation and properties of a papillomavirus infectious intermediate and its utility for neutralization studies. Virology 2014; **449**: 304–16.<br>3. Kizima L, Rodríguez A, Kenney J, et al. A potent combination microbicide that targets SHIV-RT, HSV-2 and HPV. PLoS One 2014; **9**: e94547.<br>4. Levendosky K, Mizenina O, Martinelli E, et al. Griffithsin and Carrageenan Combination To Target Herpes Simplex Virus 2 and Human Papillomavirus. Antimicrob Agents Chemother 2015; **59**: 7290–8.<br>5. Kines RC, Cerio RJ, Roberts JN, et al. Human papillomavirus capsids preferentially bind and infect tumor cells. Int J Cancer 2016; **138**: 901–11.
in vivo	1. Roberts JN, Buck CB, Thompson CD, et al. Genital transmission of HPV in a mouse model is potentiated by nonoxynol-9 and inhibited by carrageenan. Nat Med 2007; **7**: 857–61.<br>2. Roberts JN, Kines RC, Katki HA, Lowy DR, Schiller JT. Effect of Pap Smear Collection and Carrageenan on Cervicovaginal Human Papillomavirus-16 Infection in a Rhesus Macaque Model. J Natl Cancer Inst 2011; **103**: 737–43.<br>3. Derby N, Lal M, Aravantinou M, et al. Griffithsin carrageenan fast dissolving inserts prevent SHIV HSV-2 and HPV infections in vivo. Nature Commun 2018; **9**: 3881.
clinical	1. Marais D, Gawarecki D, Allan B, et al. The effectiveness of Carraguard, a vaginal microbicide, in protecting women against high-risk human papillomavirus infection. Antivir Ther 2011; **16**: 1219–26.<br>2. Perino A, Consiglio P, Maranto M, et al. Impact of a new carrageenan-based vaginal microbicide in a female population with genital HPV-infection: first experimental results. Eur Rev Med Pharmacol Sci 2019; **23**: 6744–52.<br>3. Magnan S, Tota JE, El-Zein M, et al. Efficacy of a Carrageenan gel Against Transmission of Cervical HPV (CATCH): interim analysis of a randomized, double-blind, placebo-controlled, phase 2B trial. Clin Microbiol Infect 2019; **25**: 210–6.<br>4. Laurie C, El-Zein M, Tota JE, et al. Efficacy of a carrageenan gel in preventing anal human papillomavirus (HPV) infection: interim analysis of the Lubricant Investigation in Men to Inhibit Transmission of HPV Infection (LIMIT-HPV) randomised controlled trial. Sex Transm Infect 2021; **98**: 239–246.<br>5. Laurie C, El-Zein M, Tota J, et al. Efficacy of a carrageenan gel in increasing clearance of anal HPV infections in men: interim analysis of a double-blind randomized controlled trial. J Infect Dis 2022; **227**: 402–406.

Study Protocol

Randomized Controlled Trial Evaluating the Efficacy of Carrageenan as a Topical Microbicide against HPV Infection

Division of Cancer Epidemiology
McGill University

Revised: July 2020

Amendment tracking:

- *Updated recruitment poster and joint advertisement*: approved 8 September 2014

- *Age increase from 18-40 to 18+*: **approved 17 November 2014**

- *Remuneration increase (total of $444)*: approved 24 February 2015

	Enrollment	Follow-up						Total
Remuneration (CDN$)	Visit 1	Visit 2	Visit 3	Visit 4	Visit 5	Visit 6	Visit 7	All visits
Current	20	20	20	25	25	30	30	170
**Amended**	**60**	**40**	**40**	**40**	**50**	**50**	**60**	**340**
	+2$ for completing each weekly online calendar (104$/year)							

- *Addition of a recruitment site*: **approved 19 August 2015 -** the CISSS (Centre intégré de santé et de services sociaux) de la Montérégie-Centre – Territoire Champlain-Charles-Le Moyne (Longueuil, Quebec, Canada).
- *Change in gel-usage instructions* to apply gel before and after sexual intercourse as per new research findings: **approved 26 October 2015**.
- Warning notice to study participants (avoid using condoms made from polyurethane) and handing out (for free) polyisoprene condoms for people with latex allergy (in-kind support from CarraShield Labs Inc.): **approved 28 June, 2017**

- Modifying 'question 61.1': **approved 3 February, 2017**
  61.1 Which HPV vaccine did you receive?
  1: Gardasil
  2: Cervarix
  77: Don't remember

In light of the recent (September 2016) introduction of the Gardasil 9 vaccine (offers protection against 9 HPV types) and eventual replacement of Gardasil (offers protection against 4 HPV types) with Gardasil 9, we added a third option (*3: Gardasil 9*) to 'question 61.1'.

- Adding 'sub-questions 61.2 and 61.3' to question 61 in the enrollment questionnaire: **approved 13 November, 2017**
  61.2 How many vaccine doses did you receive?
  [Drop down selection menu: numbers 1-3 or simple choice between 1, 2 or 3]
  77: Don't know or don't remember
  61.3 When was your first HPV shot?
  [Date field: dd/mm/yyyy, and an open field]

We made similar changes to an equivalent question #42 in the follow-up questionnaire.

- Proposed minor amendment of modifying/adding question(s) in the enrollment and follow-up questionnaires: **approved 11 June, 2018**

Questionnaire	Modifications	Additions
Enrollment	Question 41:<br>*What specific brand(s) of gel lubricant(s) did you use in the past month?*<br><br>**- Remove** the word "gel" from the question.	**Add** two other options ("Divine" and "Durex") to the lubricants in Question 41.
Follow-up	Question 7 would become Question 6.3	**Add** Question 6.4:<br>How many of your sexual partners since your last survey w[illegible] partners?<br><br>**Add** Question 6.5:<br>How many of these new sexual partners were your…(list san[illegible] Question 6.3)<br><br>**Add** Question 7:<br>In total, how many sexual partners have you had since the beginning of the study?
		**Add** sub-question 22.0.1 to Question 22:<br>How often did you use the CATCH gel before or during vagi[illegible] intercourse?<br><br>**Add** sub-question 22.1.1 to Question 22.1:<br>How often did you use the CATCH gel after vaginal intercou[illegible]
	Question 28.1:<br>*What other brand(s) of gel lubricant(s) did you use since your last survey?*<br><br>**- Remove** the word "gel" from the question.	**Add** two other options ("Divine" and "Durex") to the lubricants in Question 28.1.<br><br>**Add** Question 28.2:<br>How often did you use the other brand(s) of lubricant(s) duri[illegible] sexual intercourse(s)?<br><br>**Add** Question 28.3:<br>Did you purchase the other brand(s) of lubricant(s) on Amaz[illegible]<br>[IF YES to 28.3] 28.3.1 Specify which brand(s) did you purc[illegible] Amazone.ca: ______________<br><br>**Add** [IF YES to 28] 28.4 Did you purchase the other brand(s[illegible] lubricant(s) on another online website?<br>[IF YES to 28.4] 28.4.1 Specify which brand(s) did you purc[illegible] another online website:_________________

November 7, 2018: informed McGill IRB of relocation of the Division of Cancer Epidemiology Research Nurses' Office to the current headquarters of the Gerald Bronfman Department of Oncology, Division of Cancer Epidemiology as of September 17, 2018.

Amendment to collect 20 additional samples at enrolment, submitted 23 May, 2019: **approved 10 June, 2019.**

September 5, 2019: informed McGill IRB of amendment to obtain electronic consent (e-consent) from participants as we transition to Research Electronic Data Capture (REDCap) for data collection.

December 16, 2019: data are collected and managed with REDCap tools hosted as the McGill University Health Centre.

Request to resume in-person research and addendum to English consent form (addendum during the COVID-19 public health contingency: **approved July 28, 2020.**

Study Protocol

As of February 9, 2017

Sponsor: Dr. Eduardo Franco
Division of Cancer Epidemiology, McGill University
5100 Maisonneuve Blvd West, Suite 720
Montreal, Canada, H4A 3T2

Principal Investigator: Eduardo Franco, PhD, Director and Professor
Division of Cancer Epidemiology, McGill University
5100 Maisonneuve Blvd West, Suite 720
Montreal, QC, H4A 3T2
TEL: 1-514-398-8014
FAX: 1-514-398-5002

CONTENTS

STUDY SUMMARY
1.0 BACKGROUND INFORMATION
1.1 Introduction ... 4
1.2 Preclinical Studies ... 6
1.3 Completed Clinical Studies ... 7
1.4 Current Clinical Studies ... 8
1.5 Investigational Products ... 8
2.0 TRIAL OBJECTIVES AND PURPOSE
2.1 Primary Objective ... 9
2.2 Secondary Objective ... 10
3.0 TRIAL DESIGN
3.1 Primary Endpoint ... 10
3.2 Secondary Endpoint ... 10
3.3 Duration of the Study ... 10
3.4 Recruitment ... 11
3.5 Subject Participation ... 11
3.6 Frequency of the Measurement ... 11
3.7 Measures to Minimize Bias ... 12
3.7.1 Randomization ... 13
3.7.2 Blinding ... 13
3.8 Data Collection ... 13
3.9 Discontinuation Criteria ... 14
3.9.1 Individual participants ... 14
3.9.2 Study discontinuation ... 14
4.0 SELECTION AND WITHDRAWAL OF PARTICIPANTS ...
4.1 Eligibility Criteria ... 14
4.2 Completion ... 15
4.3 Loss to Follow-up ... 15
5.0 TREATMENT OF SUBJECTS ... 15
6.0 ASSESSMENT OF EFFICACY ... 17
7.0 ASSESSMENT OF SAFETY ... 17
8.0 STATISTICS ... 18
9.0 DIRECT ACESS TO SOURCE DATA/DOCUMENTS ... 19
10.0 QUALITY CONTROL AND QUALITY ASSURACE PROCEDURES
10.1 Trial Steering Committee ... 19
11.0 ETHICS ... 19
12.0 DATA HANDLING AND RECORD KEEPING
12.1 Data Handling ... 20
12.2 Data Safety and Monitoring Committee ... 20
14.0 FINANCING AND INSURANCE ... 21
15.0 PUBLICATION POLICY ... 21
16.0 SUPPLEMENTS
16.1 Table 1: Study Prodecures According To Visit ... 22
16.2 Table 2: Sample Size and Power Calculation ... 23

17.0 REFERENCES 24

STUDY SUMMARY

**Protocol Title:**	Randomized Controlled Trial Evaluating the Efficacy of Carrageenan as a Topical Microbicide against HPV Infection
**Funded by:**	Canadian Institutes of Health Research (CIHR)
**Design:**	A randomized, placebo-controlled, double-blind trial to evaluate the efficacy of carrageenan in reducing lower genital HPV incidence and prevalence and to evaluate patient adherence as measured by behavioural characteristics assessed by questionnaires.
**Population:**	Female aged 18 and older, living in Montreal
**Study Duration:**	12 months of the product use for each participant
**Primary Objective:**	Evaluation of the efficacy of carrageenan in reducing genital HPV incidence and prevalence among young sexually active women
**Secondary Objective**	Evaluation of patient adherence as measured by behavioural characteristics assessed by questionnaires
**Study Sites:**	Montreal, Canada
**Sponsor:**	Dr. Eduardo Franco<br>Division of Cancer Epidemiology, McGill University<br>546 Pine Ave. Montreal. Canada. H2W 1S6
**Investigational Product:**	Intervention Gel (treatment) and Control Gel (placebo)
**Principal Investigator:**	Eduardo Franco, PhD, Director and Professor<br>Division of Cancer Epidemiology, McGill University<br>546 Pine Ave. Montreal. Canada. H2W 1S6<br>TEL: 1-514-398-8014<br>FAX: 1-514-398-5002
**Clinical Labs:**	Student Health Services of McGill and Concordia University

BACKGROUND INFORMATION

Introduction

In 2008, Dr. Harald zur Hausen was co-recipient of the Nobel Prize in Medicine and Physiology for establishing the causal link between human papillomavirus (HPV) infection and cervical carcinoma [1]. This award reflects the importance of the discovery of a sexually-transmitted infection (STI) as a necessary cause of cervical cancer and the enormous opportunity for public health interventions. His work paved the way for two vaccines that prevent the genotypes of HPV infection that cause most cases of cervical cancer (HPVs 16 and 18), but unfortunately this intervention is too expensive for use in developing countries [2, 3]. Furthermore, HPV vaccination is exclusively prophylactic, i.e., it will prevent infections by the vaccine-target types in women who have not yet been sexually exposed, particularly pre-adolescent girls. The vaccines are ineffective against established HPV 16/18 infections or against infections by other HPV genotypes [4]. The vast majority of sexually active women will not be targeted by vaccination and in developing countries Pap test screening has failed to prevent cervical cancer [5, 6].

Epidemiology of Cervical Cancer

Worldwide, there are an estimated 470,000 incident cases of invasive cervical cancer (ICC) annually (approximately 10% of all female cancers). ICC is the 7th leading cancer site worldwide irrespective of gender and is second among women [7]. ICC is a sentinel disease of inequity, being much more common in poor countries and in aboriginal populations in Western countries [8]. It is most common in Latin America, sub-Saharan Africa, and the Caribbean; the annual incidence rate exceeds 40 new cases per 100,000. Three-quarters of the estimated 190,000 ICC deaths globally occur in developing countries, where it is the leading cause of cancer death [9]. ICC risk is comparatively lower in western Europe and North America at less than 10 new cases annually per 100,000 women [10]. In Canada in 2008, an estimated 1,300 women were diagnosed with ICC and approximately 380 deaths were attributed to the disease. The Canadian age-standardized incidence rate is 7 per 100,000 [11]. These low rates are largely attributable to Pap smear screening programs and to low fertility rates; however, a recent trend suggestive of an increase in young women in Canada and Europe suggests a cohort effect, i.e., successive birth cohorts with increasing levels of exposure to HPV infection entering cancer risk age. Moreover, ICC takes a particularly heavy toll among Aboriginal Canadians and Hispanic immigrants, groups that experience ICC rates that are comparable to those in high-risk developing countries [8].

Epidemiology of HPV

There is overwhelming biologic and epidemiologic evidence that cervical infection by certain HPV types is a precursor event in cervical carcinogenesis [12-15]. The anogenital tract's epithelial lining is the target for infection by over 40 different mucosotropic HPV types. Genital types are classified according to oncogenic potential. HPV types 16, 18, 31, 33, 35, 39, 45, 51, 52, 56, 58, 59, 66, and 68 are of high oncogenic risk because of their frequent association with ICC and high grade cervical squamous intraepithelial lesions (HSIL), also referred to as grade 2/3 cervical intraepithelial neoplasia (CIN2/3). Oncogenic types (mostly HPV 16) are also

causally implicated in other cancers, including penile, anal, and vaginal cancers [16]. Other genital types (e.g., HPV types 6,11, 42-44, and some rarer types) are considered of low or no oncogenic risk [17, 18]. These types may cause subclinical and clinically visible benign lesions known as flat and acuminate condylomata, respectively.

Most sexually active women will probably acquire HPV infection over a lifetime. Prevalent cervical HPV infection is detected in 5%-40% of asymptomatic women of reproductive age [14]. Up to 75% of adults will be infected with at least one HPV type in their lifetime. The vast majority of these infections will be transient [19-25]. A substantial increase in risk of HSIL (CIN2/3) exists for women who develop persistent infections with oncogenic HPV types [21, 26-29].

Male Condom

A condom protects the male from direct penile contact with cervical, vaginal or rectal secretions or lesions, and also protects the female from contact with penile skin, lesions, infected semen, and discharge [30]. In vitro, condoms are impermeable to all known sexually transmitted pathogens [31]. Until recently, studies evaluating male condom use against HPV infection provided equivocal results [32]. A paradoxical effect has even been reported, such that condom use appears to increase risk of HPV infection [32-34], likely a result of higher probability of infection among partners with whom condoms are used [35-37]. New evidence suggests that when condoms are used consistently, they reduce but do not eliminate the risk of male-to-female genital HPV transmission [38].

Prophylactic HPV Vaccines

Vaccination against HPV types 16 and 18 is highly effective in preventing these infections and cervical lesions in previously uninfected females [39-42]. Two vaccines [Gardasil® (Merck) and Cervarix® (GlaxoSmithKline)] were evaluated in randomized controlled trials, and both have now been approved in Canada. Both are nearly 100% effective in preventing new infections in susceptible women naïve to vaccine HPV types [42-44]. There is now compelling evidence for universal vaccination of pre-teen girls in high-income countries [45-48]. Unfortunately, for nations where the burden of HPV and ICC is the highest, vaccination remains too expensive [2, 3, 49]. Vaccination is expected to have a major impact on the burden of ICC [50]. Nonetheless, about 30% of all ICCs are caused by types other than the two vaccine-targeted oncogenic HPV types [51]. Although HPV types 16 & 18 cause ~70% of ICC cases, and are also common in genital specimens from women without cervical lesions, other oncogenic HPV types are also very common as STIs [52, 53]. Also, there is potential for type replacement, i.e., a gradual change in the distribution of HPV types in vaccinated populations due to vacated ecologic niches as HPVs 16 and 18 are eliminated. Vaccine-induced immunity may also wane beyond 10 years. Finally, vaccination is currently recommended for females 9 to 45 years of age and males 9 to 26 years of age. There is no guarantee of protection against future episodes among women who have already acquired infections. With the exception of Canada, Australia and the UK, government vaccine programs are exclusively targeting females 12 years of age at this time [54]. However, coverage that provided for herd immunity is only observed in publically-funded, school-based vaccination programs. Coverage is poor (less than 50%) in women beyond school age. In males, HPV vaccination is even less prevalent.

The Rationale for Performing Research with the Investigational Products
As discussed in the Investigator's Brochure, evidence suggests that HPV inhibitory compounds are useful for blocking the spread of HPV. Buck et al. [55] identified *carrageenan* as a potent HPV infection inhibitor. Other than vaccination, which confers partial protection and whose immunity benefits may wane, there is no other intervention against HPV infection. Pap screening has not had the expected impact in reducing ICC morbidity and mortality in developing countries. In Canada, screening has been successful; however, it generates very high costs to society because of too frequent screening (annual versus triennial) and the need to manage and treat all precancerous lesions that are found and the fertility problems that occur post-treatment. As a primary prevention approach, a carrageenan-based strategy would minimize these negative public health consequences of screening. Previous efficacy and safety tests show that carrageenan has a desirable potential to be one of the most effective strategies against genital HPV infection and associated diseases. Enhanced compliance can be achieved by including carrageenan in condoms and/or applied by women before sex to prevent HPV infection.

At this time there is no way to treat cervical HPV infections. If carrageenan is proven to be effective in preventing and/or clearing HPV infection, this will have enormous and immediate public health implications. With broad spectrum anti-HPV activity, it will be a useful adjunct to HPV vaccination in developed countries and as a primary means of preventing HPV infections and ultimately ICC in developing countries. Also, compared with HPV vaccination, use of a carrageenan gel may be more cost-effective. Despite being required to apply the gel on an ongoing basis, its costs are substantially less and there is a strong possibility that it will protect against all types of HPV. In addition, a discreet method of prevention such as this would make it empowering for women who are unable to refuse sex due to cultural, social, or financial arrangements [60].

The two HPV vaccines available in Canada only protect against a maximum of 4 types of HPV (6, 11, 16 and 18) and we are interested in investigating the effect of carrageenan against all HPV types. Considering that uptake of vaccination in the "catch-up" age range (19-45 years) has been very low so far and because data is currently insufficient to recommend either for or against vaccination among individuals in this age range, we expect only a small proportion of trial participants (<10%) will have received the HPV vaccine. Therefore, we do not anticipate any issues regarding vaccine uptake and comparability between groups.

Preclinical Studies
Previous clinical studies state that HPV inhibitory compounds (deliverable as topical microbicides) are useful for blocking the spread of HPV [55-57]. Buck et al. [55] identified *carrageenan* (an inexpensive gelling agent that is non-toxic and safe in animals and humans) as a potent HPV infection inhibitor. Importantly, inhibition occurred against all high and low oncogenic risk HPV types, including 6, 11, 16, 18, 31 and 45; the latter four types being responsible for over 80% of ICCs worldwide [58]. There has been interest in carrageenan as a vaginal microbicide targeting HIV and herpes viruses, but cell culture tests have found that it is a thousand times more effective against HPV than against HIV. Recently, carrageenan was shown to inhibit genital transmission of HPV in mice and its evaluation in efficacy studies in humans is

now warranted [57]. These studies provide the evidence base to suggest that carrageenan may be effective against HPV.

Completed Clinical Studies

As described in the Investigator's Brochure, investigators developed and patented Carraguard (a λ-carrageenan derived microbicide gel) for a large $40 million HIV prevention trial funded by the Bill and Melinda Gates Foundation and the U.S. Agency for International Development [55, 67, 68]. Previous safety and acceptability trials of Carraguard included participants from the U.S., Thailand, Chile, Australia, Dominican Republic and South Africa. Participants found the gel pleasant or neutral in feel and considered extra lubrication to be an advantage [69, 70]. The carrageenan-based gel proposed for our trial is essentially identical to Carraguard. Therefore, we expect that it will also be well received by most participants in our study. Carraguard was not found to be efficacious against HIV [68], a finding that does not diminish the expectation that a carrageenan-based intervention is likely to prevent HPV infections since the inhibitory activity of this compound against HPV is 1000 times stronger than that for HIV [55]. Investigators from the Carraguard trial found no increased risk in other (non-HPV) reproductive tract infections in the carrageenan arm compared to placebo [68]. The excellent safety and acceptability findings from the HIV study strengthen the case for a study aimed at HPV prevention.

The phase I preliminary safety study determined the safety and acceptability of vaginal gel formulation PC-503 among low risk, abstinent women. The active ingredient was 2% pharmaceutical grade lambda carrageenan, a sulphated polymer that is generally recognized as safe by the US Food and Drug Administration. 35 women in five sites applied 5 ml of the PC-503 gel vaginally once a day for 7 days while abstaining from sexual intercourse. Following product use, five reported mild symptoms including "bladder fullness," "genital warmth," or discomfort, and lower abdominal pain, and one had moderate pale yellow cervical discharge. Using the Nugent criteria, three women had bacterial vaginosis (BV) before and after use; three had BV before but not after, and two had BV after but not before. Most of the women found PC-503 to be pleasant or neutral in feel and smell and considered extra lubrication to be an advantage; however, one third found it to be messy. Vaginal use of PC-503 gel did not cause significant adverse effects in a small number of low risk, sexually abstinent women.
The study of acceptability of a large scale Phase III testing in South Africa provides valuable insight into desirable product characteristics among a select population group where these trials are conducted. In general, Carraguard was found to be acceptable among the HIV-positive women and men in this study, with very few participants reporting side effects after use and most expressing generally favorable opinions about the product. However, certain product features, most notably gel volume, may require reformulation (e.g. higher viscosity) to optimize product acceptability in certain populations or settings. In this recent South African Carraguard trial, women were asked to apply carrageenan or placebo gels vaginally using a pre-filled, single-dose, disposable applicator every time before sex. Compliance in this trial was very low. Women reported using the gel only 44% of the time, and just 10% said they always used it before sex [68, 97]. Among all interviewed at baseline (N = 6202), only 34% reported having used a condom at their last sexual act [68]. Other African trials of vaginal microbicide gels have observed compliance rates >70% [98, 99].

Previous safety and acceptability trials report the gel as being safe and generally well received [69-71]. In a separate trial evaluating the safety of 2% ι-carrageenan applied intravaginally once a day for seven days, the gel was not associated with any significant irritation [71]. Only 1/25 women in this trial had evidence of inflammation, which was asymptomatic and minor and was only discovered via colposcopic examination. One other participant was observed to have a small vaginal abrasion. Finally, formulations of carrageenan were not associated with vaginal flora changes when used for up to one year [72, 73] and direct application to the penis was not associated with any irritation [74].

Current Clinical Studies
No clinical trials on this topic are currently registered in the clinical trials registry (http://clinicaltrials.gov/ct2/search). As far as we know, no previous trial has evaluated the efficacy of carrageenan against HPV in human subjects. We propose a randomized controlled trial because there is a genuine public health need to assess the efficacy of carrageenan against HPV in humans. The trial will be conducted in compliance with the protocol, Good Clinical Practice (ICH/GCP), and the applicable regulatory requirements of Natural Health Product Directorate (NHPD) and Health Canada. The study population will consist of females aged ≥18 years, living in Montreal. A volunteer sample will be used, as a population-based study would have extremely low response and compliance rates. Willingness to return for follow-up will be greatest among committed volunteers. A volunteer sample also maximizes internal validity due to improved protocol adherence and provision of more accurate information on risk factors and sexual histories.

Investigational Products
Two commercially available products, Intervention and Control Gels, are proposed for this trial. These types of viscous gels were ranked highest by female university aged women in a feasibility study we conducted. They are both clear, odourless, tasteless, and have a similar viscosity. Both gels are water based and latex condom compatible. The most important difference between the two gels formulation is that one contains carrageenan and the other does not.

Intervention Gel

The Intervention product is manufactured in a U.S. FDA GMP-compliant facility in Atlanta, Georgia, U.S.A. In keeping with GMP requirements, the manufacturing process is under continuous quality control with Certificates of Analysis documented for each ingredient. The product is fragrance, paraben and glycerin-free, making compatible with sensitive skin. The formula is primarily water-based, making it latex safe. All ingredients are edible, making it oral sex compatible.

Components: 55.5% Purified Water, 37.76% Propylene Glycol, 5% Saccharin, 1.67% Carrageenan, <0.3% Phenoxyethanol, <0.1% Chlorphenesin, <0.01% Citric Acid

Divine Corporation has marketed its personal lubricant to the general consumer market for the past ten years. Stability testing conducted on three year aged samples by an independent laboratory has shown the product to be highly stable. Consumer feedback has been positive and

the company reports no returns for skin irritation or product stability problems in the approximately ten years of consumer marketing.

The Intervention Gel has been personally tested by licensed U.S. physicians and these physicians recommend this product to their patients. Divine Corporation maintains signed affidavits from physicians which state their personal and professional endorsement of the product.

Control Gel

The Control Gel is manufactured in a U.S. FDA GMP-compliant facility in Chatham, California, U.S.A. and bottled in a FDA GMP-compliant facility in Atlanta, Georgia, U.S.A. In keeping with GMP requirements, the manufacturing process is under continuous quality control with Certificates of Analysis documented for each ingredient. This product is fragrance free and compatible with all skin types. The formula is primarily water-based, making it latex safe. All ingredients are edible, making it oral sex compatible.

Components: 35% Purified Water, 35% Propylene Glycol, 18%Glycerin, 1% Tocopheryl Acetate (Vitamin E), 5% Cellulose Gum, 5% Hydroxyethylcellulose, <.33% Tetrasodium EDTA, <.33% Potassium Sorbate, and <.34% Sodium Benzoate

The ingredients for the Control Gel are used in varying proportions in numerous off-the-shelf personal lubricants sold as cosmetics.

Intervention Gel
Treatment with carrageenan-containing vaginal gel, self-applied every other day (whether or not participants have intercourse) for the first month and prior to each act of vaginal or anal intercourse during the entire study period.

Control Gel
Treatment with placebo vaginal gel, self-applied every other day (whether or not participants have intercourse) for the first month and prior to each act of vaginal or anal intercourse during the entire study period.

Prior to vaginal and/or anal intercourse, participants will be asked to apply the study gel either directly inside their vagina, genitals, and anal and perianal area using their fingers. Both active and placebo are packaged in a plastic bottle with a desk closure that can be operated with one finger. This frees one hand to perform the application. During intercourse, additional lubricant can be applied as desired. During the first month, participants will be asked to apply the gel both inside their vagina, as well as on to their genitals. A study nurse will provide instructions on how to safely apply and remove the gels. Study participants will be asked to continue using the assigned intervention for the complete follow up period (1 year) independently of any other methods of contraception and/or STI prevention (e.g., condoms). Compliance will be measured via self-administered questionnaires during follow-up. To reduce the possibility that gel use could interfere with HPV DNA testing, participants will be asked to abstain from vaginal and oral sex and gel use for 48 hours prior to a scheduled clinic visit.

TRIAL OBJECTIVES AND PURPOSE

Primary Objective

To evaluate the efficacy of carrageenan in reducing genital HPV incidence and prevalence among young sexually active women

Secondary Objective

To evaluate patient adherence as measured by behavioural characteristics assessed by questionnaires

TRIAL DESIGN

The study will be conducted in compliance with the protocol, Good Clinical Practice (ICH/GCP), and the applicable regulatory requirements of Natural Health Product Directorate (NHPD) and Health Canada.

This is a Phase 2b randomized, placebo-controlled trial to evaluate the efficacy of carrageenan in reducing genital HPV incidence and prevalence and to evaluate patient adherence as measured by behavioural characteristics assessed by questionnaires.

Primary Endpoint

The primary outcome 1 is presence of (i) a newly detected vaginal infection in someone who was HPV negative at enrolment or (ii) HPV types other than those observed at enrolment. The primary outcome 2 is clearance of infections with HPV types observed at baseline. HPV DNA detection and genotyping of vaginal samples will be done by the PGMY polymerase chain reaction protocol.

HPV detection and typing will be done via the well-established PGMY PCR protocol coupled with the linear array method, commercially available from Roche. This test permits testing and typing for 37 genital types of HPV (6, 11, 16, 18, 26, 31, 33, 35, 39, 40, 42, 45, 51, 52, 53, 54, 55, 56, 57, 58, 59, 61, 62, 64, 66, 67, 68, 69, 70, 71, 72, 73, 81, 82, 83, 84, 89) [89]. As an a priori rule to guide the exploratory statistical analyses, HPV types 16, 18, 31, 33, 35, 39, 45, 51, 52, 56, 58, 59, 66, and 68 will be considered as of high oncogenic risk, whereas the remaining types will be considered low-risk, i.e., non-oncogenic [28]. All assays will be done in Dr. Coutlée's laboratory, one of the most experienced and specialized in HPV diagnostics worldwide. He was one of the lead investigators in the team that validated Roche's PGMY linear array technique for clinical use.

Secondary Endpoint

Participants will self-complete a computerized questionnaire at each clinic visit. This questionnaire will measure known and potential HPV risk factors, compliance with the intervention, and monitor their safety and acceptability. To assess adherence, participants will be asked to log into a secure website on a weekly basis to update their sexual activities and study gel use using an electronic calendar.

Duration of the Study

Study Protocol

Study participants will be asked to continue using the intervention that they are assigned for the complete follow up period (1 year) independently of any other methods of contraception and/or of protection against STIs (e.g., condoms). Compliance will be measured via self-administered questionnaires during follow-up.

HPV infection status will be measured at baseline (enrolment/ time 0), 14 days and 1, 3, 6, 9 and 12 months after enrolment. A one-year follow-up period was chosen to allow sufficient opportunity for HPV transmission. Although a longer follow-up would reduce our effective sample size, the burden to participants would be much greater and would likely result in a lower compliance rate.

Recruitment

Our base for recruitment will be the McGill and Concordia University Health Services Clinics. The clinics provide medical care year-round to full-time students. Our team has conducted three studies among women attending these clinics [34, 91, 92, 95, 96]. The first was a cross-sectional study of risk factors for prevalent cervical HPV infections [34]. Of 1300 eligible women attending these university health clinics for routine Pap testing over a 13-month period, 500 were randomly sampled and approached, 489 (98%) agreed to participate, and 411 (82%) completed and returned the questionnaire. In the McGill-Concordia cohort study [91], women were followed for a 24-month period for observation of the acquisition and persistence of HPV. A total of 635 female participants were recruited in a 27-month period. The third study (currently underway) is the HITCH (HPV Infection and Transmission Among Couples through Heterosexual activity) cohort study [92, 93]. To further our understanding of the transmissibility of HPV, this study enrols young heterosexual couples that are followed for HPV types not present at baseline. Recruitment began in 05/2005 and 882 participants (429 couples and an additional 24 males) enrolled as of 06/2010.

Recruitment will be bolstered through campus-wide appeals (e.g. posted notices, e-mails to student lists). Additional efforts will include mail-outs to students living in residence, presentations to students in professional schools (e.g. medical school), and information booths at student activities. Word-of-mouth will also be used for 'snowball' sampling. Based on our previous experience, we expect to recruit about 130 to 200 participants per year, which would permit accruing the complete sample of 465 subjects in just over 3 years. Also, given our experience recruiting subjects in university settings and that subject recruitment resources are already in place, we do not anticipate any major issues with recruitment.

Subject Participation

The proposed study will restrict participation to young women because most studies indicate a sharp decrease in prevalence after age 30 [77-80]. HPV acquisition is high among young adolescent and adult women, with an estimated 46% acquiring at least one HPV type within 3 years of sexual debut [81].

The Frequency of the Measurement

Vaginal specimens will be obtained for the above 7 time points during clinic visits (i.e., enrolment/time 0, 14 days and 1, 3, 6, 9 and 12 months after enrolment). Women will be asked to abstain from intercourse, oral sex, and gel use a minimum of 48 hours before specimen

collection. This will minimize the risk of contamination with residual male epithelial cells, urethral secretions, and or/semen [84]. The reason we plan to test subjects on day 14 and at month one is to evaluate whether carrageenan is also effective at clearing existing HPV infections. Since the majority of infections detected at baseline will be transient, it is important to evaluate this outcome shortly after enrolment.

Frequent measurement of HPV status is also necessary to minimize error in estimating time of infection and in detecting short-duration transient infections. Because no study can measure HPV status daily, HPV incidence will be interval-censored (i.e., infection date will be known to have occurred sometime between the last negative and first positive test). The need for precise measurement must be offset by the need to minimize participant burden and financial costs.

The First Clinic Visit
At the first visit, a research nurse will provide a participant with instructions on how and when to apply the vaginal study gel and on the self-collection of vaginal HPV specimens. The research nurse will also show her how to use the computerized survey. She will then collect her first vaginal HPV specimen, conduct a urine pregnancy test, an HIV test, and complete the first survey in a private room at the clinic. The QuickStep® Plus HCG Combo Test Kit will be used to test for pregnancy. Human chorionic gonadotropin (hCG) is a glycoprotein hormone secreted by the developing placena shortly after implantation and can be detected as early as 6 to 15 days after conception using this test. The INSTI HIV-1 Rapid Antibody Test will be the HIV test used to screen patients in this trial. This test requires only a small amount of capillary blood for testing (50 uL) obtained via a finger-stick (or finger-prick) procedure using a lancet. The research nurse will be available at all times should she need help. A participant will then be given an access code so that she can fill out subsequent surveys through a secure Internet website. She will also be assigned an individual code for the duration of the trial (this code will be used to match her to an intervention). Before leaving, she will be provided with one month's supply of gel. All participants will be provided with information cards to distribute to their partner(s). This first visit will last about one hour.

Subsequent Clinic Visits
A participant will visit the clinic at 14 days, 1 month, 3 months, 6 months, 9 months and 12 months after the first visit. She must abstain from vaginal intercourse, oral sex, and gel use for at least 48 hours prior to each visit (as indicated above). At the clinic she will briefly meet with the research nurse who will ask her to collect a vaginal specimen for HPV testing and complete a follow-up survey in a private room. At her one month visit and every visit thereafter, the research nurse will provide her with a 3 month supply of study gel (i.e., enough to last until the next visit). Each visit will last about 15 minutes. Subjects will only receive an exit Pap exam if they are ≥25 years of age and if they have not received an exam within the past three years. The Natural Health Products Directorate will be notified of all serious adverse events (expected and unexpected) that are reported or detected during the trial in an expedited manner.

Weekly Electronic Calendar

To assess adherence, participants will be asked to log into a secure website on a weekly basis to update their sexual activities and study gel use using an electronic calendar.

Measures to Minimize Bias

Table 1 shows the study procedures according to study visit. Assignment of the intervention will be done at McGill University's Division of Cancer Epidemiology (MDCE) coordination centre via computer-assisted block randomization with randomly variable block sizes. Randomization and supervision of the study will be carried out by the Study Director Dr. Mariam El-Zein.

Randomization

Interested individuals will be screened over the telephone or in person. Eligible and interested women will attend their enrolment visit, at which time the nurse will obtain informed consent and instruct the participant on vaginal gel use and on the self-collection of specimens. The participant will receive one month's supply of gel and will provide the first specimen. A random set of numbers will be assigned to the treatment gel and a different random set to the control gel. Each participant will be assigned an individual code. This code will be used to match the participant to the study arm. Finally, the nurse will provide information about HPV infection and sexual health.

Blinding

To ensure participant blinding, the two gels (carrageenan and placebo) and their containers will look and feel identical. The two commercially available products for this trial are water based, condom compatible and are actively marketed in the U.S. consumer market as personal lubricants following FDA guidelines for cosmetics. The only important difference between the two gels' formulations is that one contains carrageenan and the other does not. They are both clear, odourless, tasteless, and have a similar viscosity. They will both be bottled in identical containers and labelled the same. Only an encoded lot number printed on the bottom of the bottle will distinguish the two.

The success of blinding will be evaluated at 6 and 12 months. Despite the risk of compromising blinding (by asking patients to guess their assignment) and lack of a "gold standard" technique to measure blinding, we believe this information is still very useful and worth the risk (e.g., if the majority guess their assignment correctly it would suggest that blinding was ineffective).

Data Collection

Self-collection methods have been shown to be valid for research and clinical purposes, and acceptable to women [85-87]. The instructions for self-collection of vaginal specimens will follow those of the validated protocol of Gravitt et al. [87]. Women will be instructed to gently insert a Dacron™ swab into the vagina until physically it cannot go any further (at least 5 cm), then to rotate the swab inside the vagina for three full rotations. The research nurse will agitate the swab in a plastic vial containing liquid preservative (Preservcyt™, Hologic Co, Marlborough, Mass.), press it against the side of the vial to express any remaining fluid, then discard the swab. PreservCyt adequately preserves exfoliated specimens for DNA, RNA, and protein analyses. The swab sample in PreservCyt solution will be kept at in a refriерator at 4°C pending transfer to Dr. Coutlée's laboratory. Samples will be batched and transported every 45 days maximum, sooner if there are a large number of sample vials. After centrifugation at 13,000 *g* for 15 min at 22°C, the supernatant will be discarded, and the cell pellet left to dry and resuspended in 300 µL of 20mmol/L Tris buffer (pH 8.3). Finally, DNA will be purified using a Master- Pure Kit (Epicentre) [88] and tested in each PCR assay.

Discontinuation Criteria

Individual participants

A participant may be discontinued early from the study for any of the following reasons:

- She becomes pregnant
- She considers it to be in her best interest or for personal reasons
- A physician considers it to be in the participant's best interest because of safety reasons and/or for the well-being of the participant.
- She cannot be reached for follow-up visits after 15 months has passed from the date of randomization

Study discontinuation

The trial will continue until its planned completion, unless:

- McGill IRB decides to discontinue the trial due to noncompliance with protocol/regulations; or, interim monitoring of patient safety data, treatment efficacy data, logistics information such as patient accrual rates, and quality assurance information suggests that study should be stopped for ethical or scientific reasons
- Health Canada terminates the trial (i.e., it withdraws approval of the CTA)

SELECTION AND WITHDRAWAL OF SUBJECTS

Eligibility Criteria

- We plan to recruit female aged 18 and older, living in Montreal. Eligible subjects must: plan to remain in Montreal for at least the next year; have had vaginal sex with a male partner during the last 3 months and expect that they will do so again in the next 3 months, regardless of whether or not the male partner(s) will change; not currently in a relationship that has lasted longer than 6 months; be willing to follow study instructions; understand French or English; be willing to comply with follow-up for at least 12 months; have an intact uterus; have no history of cervical lesions/cancer or genital warts; not be pregnant or planning to immediately become pregnant and not currently breast-feeding; not had a recent (within the last 6 weeks) pregnancy, abortion, or genital surgery; be using a medically acceptable method of contraception and intend to use it for the duration of the trial; have no HIV infection; have no known allergy or hypersensitivity to vaginal lubricants; and have no allergy to all of the ingredients of the study product or placebo. Since there are many HPV types, this trial will not exclude women who have a detectable HPV type upon enrolment as these women could still become infected with another HPV type. In our previous study, women infected with one HPV type at baseline were more likely to acquire a new type [76]. Finally, individuals who have participated in any research studies (past 3 months) related to HPV or cervical cancer; or studies that require taking medications or supplements, undergo medical tests or procedures, or undertake dietary or exercise regimens will not be eligible to participate.

Completion

A participant will be considered to have completed the study after she has completed 12 months of follow-up (using either carrageenan or placebo gel), and after her final set of data has been collected and entered.

Loss to Follow-up

Since we plan to offer participants a generous incentive to remain in the study, the rate of loss to follow up is expected to be low. In the HITCH cohort study, female participants currently receive an incentive of $50 for each clinic visit to ensure adequate enrolment and retention during follow up. We will offer an incentive of between $40 and $60 per clinic visit ($60 for visits 1 and 7, $40 for visits 2-4, and $50 for visits 5-6), which is less than for HITCH because the latter includes a blood sample collection, as well as oral and hand specimens. Participants will also receive $2 for the completion of each weekly electronic calendar (paid at each clinic visit). In the McGill-Concordia cohort study [91], cumulative attrition was 10% at 12 months (using a $10 incentive). Similarly, retention in HITCH cohort study has been excellent; to date the attrition rate is 6% at 12 months. We anticipate that the rate of loss to follow-up for the current study will be between 6% and 10%, and will not be differential.

TREATMENT OF SUBJECTS

As indicated previously, two commercially available products are proposed for this trial: Intervention Gel (treatment) and Control Gel (placebo). Both of these gels are water based, oil free and condom compatible. Safety and tolerance of the gels has been demonstrated through ten years of consumer sales.

Subjects randomized to receive the carrageenan-containing vaginal gel will be asked to self-apply it every other day (whether or not participants have intercourse) for the first month and prior to each act of vaginal or anal intercourse during the entire study period. Similarly, subjects randomized to receive the placebo vaginal gel will be asked to self-apply it every other day (whether or not participants have intercourse) for the first month and prior to each act of vaginal or anal intercourse during the entire study period. Both gels come in a plastic bottle with disk cap. Such bottles and closures are commonly used for similar medium-viscosity gels such as suntan lotions, shampoos and conditioners. For application, approximately 10mL of the personal lubricant provided is dispensed into the hand and applied gently to the genital surfaces and the vagina. The gel can also be applied to the penis, the perianal tissue, the anus or other mucous membrane. After direct application has occurred, contact between the individuals is initiated with the lubricant located between the individuals. The gel can also be used as a lubricant in combination with a prophylactic device, such as a condom or a diaphragm. When used with a condom, the gel may be applied directly to the condom prior to vaginal intercourse. When sexual activity ceases, the gel can easily be removed using lukewarm water, as the formulation is water based.

HPV infection status will be measured at baseline (enrolment/ time 0), 14 days and 1, 3, 6, 9 and 12 months after enrolment (table 1). Study participants will be asked to continue using the intervention that they are assigned for the complete follow up period (1 year) independently of any other methods of contraception and/or of protection against STIs (e.g., condoms).

Compliance will be measured via self-administered questionnaires during follow-up. Although a longer follow-up would reduce our effective sample size, the burden to participants would be much greater and would likely result in a lower compliance rate.

The need for participants to use the gel prior to vaginal intercourse may pose some compliance issues. In the recent South African Carraguard trial, women were asked to apply carrageenan or placebo gels vaginally using a pre-filled, single-dose, disposable applicator every time before sex. Compliance in this trial was very low. Women reported using the gel only 44% of the time, and just 10% said they always used it before sex [68, 97]. Among all interviewed at baseline (N = 6202), only 34% reported having used a condom at their last sexual act [68]. We expect that our results will be much better. First, other African trials of vaginal microbide gels have observed compliance rates >70% [98, 99]. Second, we will restrict participation to women who are willing to comply with study instructions. Third, the gel will not be applied with an applicator, but in the same fashion that common sexual lubricants are used. Fourth, we will promote the gels as being fun rather than an interference with the spontaneity of sexual activity; the belief that lubricants enhance pleasure was associated with their use among young women [100]. Fifth, participants will be provided with information pamphlets and will be able to share this information with their partner(s). Sixth, participants will be telephoned weekly for the first month and monthly after that and will be reminded to use the gel with condoms at each sexual intercourse act. During these conversations, participants may ask any questions or discuss any concerns regarding use of the gel, or the trial itself. Participants will also be asked if they need more gel and whether they have experienced any adverse events (either study or non-study related). Finally, participants with cell phones will also be sent weekly emails or text messages kindly reminding them to remain compliant.

It is reasonable to expect frequency of gel use to be high during the initial phase of our trial. Whether we should expect participants to remain compliant for the entire study duration is unknown. The current trial will provide us with an answer to this question, which will assist us in the conduct of future studies.

Additional indirect evidence to support our expectation for compliance comes from our HITCH cohort study. At enrolment, 91% of HITCH participants reported using condoms and 37% reported using them most of the time or always (>75% of the time). Although issues are known to exist surrounding the self-report of condom use [101], this method of report for condom use has previously been shown to be a valid indicator of STD risk among college students and adolescents in the US [102, 103]. Microbicides are similar to condoms in the sense that both must be applied prior to intercourse, and frequent condom use is a predictor of lubricant use [100].

Previous safety and acceptability trials also demonstrate that carrageenan vaginal gels are safe and generally well tolerated [69-74]. We consider the present study a phase IIb RCT, since to our knowledge it is the first such study to test the potential efficacy of carrageenan as a vaginal gel to prevent HPV transmission. If this initial study demonstrates that the intervention is efficacious we intend to launch a much larger phase III investigation involving multiple centres across Canada and internationally, using our collaborative ties with HPV and ICC prevention teams elsewhere. Data from the present trial will provide important insights into the challenges and

determinants of protocol adherence and will provide more realistic estimates of effect size. A future study should also examine other means of delivering the intervention for greater impact, e.g., using condoms packaged with carrageenan-containing lubricants, and of examining transmission in more detail, e.g., by recruiting women and their partners and testing for HPV infection in both to assess whether there is an impact in reduced transmission within couples. Likewise, in a future study we intend to assess health services delivery and quality of life measures. Conducting such a complex phase III study at this stage would have been prohibitive in terms of cost and logistics, especially without having solid proof of concept that the intervention is efficacious in at least one clinical study.

ASSESSMENT OF EFFICACY

Calculation of carrageenan's efficacy will be done by testing the null hypothesis of no difference in time to HPV infection (i.e., infection with an HPV type not present at baseline) between treatment groups with the log rank test. HPV infection date will be considered the midpoint between the first visit date that a new HPV type is detected and the previous test date at which time the new HPV type was not present. Time to HPV infection will be defined as the difference in days between the calculated HPV infection date and the enrolment date plus one. We will use a Cox proportional hazards regression model to estimate the hazard ratio and 95% CI of HPV infection for the treatment versus placebo group. To evaluate effectiveness of carrageenan gel in our study population we will perform our analyses according to the intention-to-treat approach (i.e., including all participants who were randomized and received at least one-month's supply of vaginal gel). To evaluate efficacy we will also use the according-to-protocol approach (i.e., including only "adherent" participants who complied with the study protocol). A participant will be considered adherent if they report gel use as recommended (within one hour of intercourse) in >50% of all intercourse acts. In our interim analyses, we will allow for time-varying adherence, defined as adherence since the last questionnaire.

We will also use actuarial techniques to measure clearance of infections with HPV types present at enrolment according to the intervention. Time to clearance and hazard ratios of clearance will be calculated as above for participants with prevalent HPV infections at enrolment. Because of randomization the rates of type-specific HPV infections will be comparable between study arms at enrolment. Such analyses will use intention-to-treat and according-to-protocol approaches.

The proportion of participants remaining adherent will be calculated and compared between groups (intervention vs. control) and for all participants combined. We will use the chi-square test to compare adherence between intervention and control groups at each follow-up visit (adherence since the last questionnaire), as well as overall adherence (adherence from month 0 to month 12).

ASSESSMENT OF SAFETY

Adverse event reporting

We will make use of the Female Genital Grading Table for Use in Microbicide Studies and the Division of AIDS Table for Grading the Severity of Adult and Pediatric Adverse Events (Version 1.0, December 2004) for grading adverse events (AE). In cases where an AE is covered

in both tables, the Female Genital Grading Table will be the grading scheme utilized. At the baseline visit we will be administering a medical history questionnaire to gather information on underlying health conditions. Subjects will be advised to notify a research nurse immediately of any adverse events, at which point they will be contacted and triaged accordingly. In addition, subjects will be asked to report adverse events, or any recent medical visits at each follow-up visit.

The data safety and monitoring committee will meet periodically during the course of the trial to review the data. This committee will review the accruing trial data and make recommendations regarding safety issues or reasons that may force early trial termination (e.g., early evidence of a definite efficacy due to the intervention). Members of this committee will be independent of the trial; at this point we are awaiting the IRB's recommendation with respect to the possible committee membership. Members will have expertise in statistics, HPV prevention, the conduct of clinical trials, and ethics/law.

STATISTICS

Assumptions for sample size calculations were based on our experience in conducting two cohort studies of the epidemiology of HPV infection in university students in Montreal: the McGill-Concordia cohort study [91] and the HITCH cohort study of couples [92, 93]. Using estimates from these studies specific for the subset of women who will be eligible for the trial, we derived a cumulative incidence proportion of infection at 12 months as 19.5% (average from these 2 studies). Similarly, clearance of baseline HPV infection (any type) was estimated from these studies to be 38% after 12 months. For the sample size calculations we used the technique of Dupont and Plummer [94] and the hazard rate estimates of acquisition and clearance. This approach is consistent with the aim of studying outcomes at different time points via survival analysis (i.e., log-rank test and Cox proportional hazards regression). Based on the strong inhibitory properties of carrageenan demonstrated in vitro and in animal studies [55, 57] and in view of the expert opinion of Dr. John Schiller, the senior investigator at the US National Institutes of Health who made the discovery [55] of carrageenan's strong HPV inhibitory properties (appended letter), we assumed that the intervention could translate into preventive effect sizes of 50%-75%. As additional parameters we specified 80% power to answer both questions (preventing acquisition and accelerating the rate of clearance of existing ones) with type one error of 0.05 and 2-sided hypothesis.

Assuming losses to follow-up of about 10% at one year (the rate we observed in our two aforementioned cohort studies) and baseline infection prevalence of about 50% (also based on estimates from our two previous studies) [91, 92], the total sample size required to evaluate our primary aim 1 (prevention) for effect sizes of 50%, 60% and 75% was determined to be 463, 302 and 174, respectively, for both arms. Similarly, assuming a clearance rate of about 40% in controls [91, 92], the total sample size required to evaluate our primary aim 2 (clearance) based on the above effect sizes was determined to be 388, 268 and 168, for both arms, respectively. To permit verification of the study's objectives for a 50% effect size with sufficient power at the end of the one year follow-up period, we propose to recruit 465 subjects. Table 2 reveals the effect that varying parameter estimates and effect sizes has on the sample size required to satisfy both of the study aims (prevention and clearance).

To decide whether or not the trial should terminate early, the available data will be analyzed annually. The type 1 error for concluding efficacy will be controlled by the Lan-Demets spending function [104] with O'Brien and Fleming type boundaries [105]. The Lan-Demets method offers us flexibility to analyze the data either sporadically, or at equal intervals. An independent data safety monitoring board will review the interim analysis results. We have sought advice to McGill University's IRB concerning the composition of this board. Members will likely be nominated by the IRB, outside of our purview.

DIRECT ACESS TO SOURCE DATA/DOCUMENTS

It is specified in the protocol that the investigator will permit trial-related monitoring, audits, IRB/IEC review, and regulatory inspections, providing direct access to source data/documents.

QUALITY CONTROL AND QUALITY ASSURANCE PROCEDURES

Trial Steering Committee

This committee will provide overall supervision of the trial, will ensure that it is being conducted in accordance with the principles of good clinical practice and the relevant regulations, and will make decisions about continuation or termination of the trial or substantial protocol amendments. The Chair will be Dr. Alex Ferenczy, Professor of Gynaecologic Pathology at McGill University and one of the world's leading experts on HPV-associated diseases. He has not been involved with the investigators in designing this study and brings considerable expertise as previous consultant to the HPV vaccine trials conducted by industry. In that capacity he acquired substantial experience regarding the definition of HPV prevention endpoints and other outcome measures. Dr. John Schiller, a Senior Scientist with the US National Cancer Institute and the lead investigator who made the discovery of the anti-HPV properties of carrageenan and conducted the pre-clinical studies, will also serve in the committee. We will invite a leading Canadian biostatistician. Additional members include the PI (Dr. Franco) and Study Director (Dr. Mariam El-Zein). This committee will meet twice a year to review progress and to advise on issues related to protocol adherence, endpoints, and statistical analysis.

As a phase II trial of initial verification of clinical efficacy, health service research issues will not be directly addressed by the proposed study. The purpose of this trial is to evaluate the efficacy of carrageenan delivered as a vaginal gel against HPV infection. Should our trial demonstrate efficacy, its findings will be instrumental in orienting initial estimates of cost-effectiveness by our team or others. Our post-hoc findings related to compliance, costs, and per-protocol efficacy will be important for such subsequent calculations. To minimize costs we have not included in the study the expertise and resources to permit such analyses as part of the core objectives. Likewise, traditional quality of life measures, such as the SF-36 questionnaire, will not be used in the interest of decreasing the probability of non-compliance and losses to follow-up. Other studies have already attempted to explore the issue of quality of life in individuals diagnosed with HPV [90].

ETHICS

The lubricant gels being proposed for this trial are water based, safe, and are available over the

counter without a prescription. However, there is some concern that participation in the trial might inappropriately convince women who have not had the HPV vaccine that it is unnecessary. Despite the fact that previous efficacy and safety tests demonstrate that carrageenan has the potential to be one of the most effective prevention strategies against genital HPV infection (all types), vaccination targets the HPV types that pose the greatest cancer risk to most young women. Given the availability and affordability of the vaccine in Canada, there are ethical considerations in conducting a trial in a population which, unlike women in the developing world, does have access to the HPV vaccine. The CIHR Public, Community & Population Health Peer Review Committee emphasized that it is important to inform women that vaccination is available, and to re-iterate the availability of vaccination again at the end of the study. While it is necessary and appropriate to encourage women who have not been vaccinated prior to enrollment to obtain the HPV vaccine on exit from the study, it is important to remember that both vaccines (Gardasil™ and Cervarix™) are exclusively prophylactic; and considering that women who participate in this trial are highly sexually active, there is a strong chance that most will have been exposed to the HPV vaccine target types prior to enrollment.

This research project has received the ethical approval from the Institutional Review Board of the Faculty of Medicine, McGill University (conditional on approval from Health Canada). The members of the Data Safety and Monitoring Committee will have expertise in the conduct of clinical trials and ethics/law. Each member will pay close attention to any safety and ethical issues that may influence the design and/or analysis of the study. The Trial Steering Committee, along with all the participating investigators, will provide overall supervision of the trial.

DATA HANDLING AND RECORD KEEPING

Data Handling

Study and data management will be facilitated through the use of a secure, password-protected web-based administrative database to record and manage study procedures. The database will be used to record participant and clinic visit information; review due, overdue and completed clinic visits and surveys; and for exporting data. It will only be accessible from specific IP addresses.

Data Safety and Monitoring Committee

The chair of the Data Safety Monitoring Committee for this study is Anthony B. Miller, MD, PhD (Professor Emeritus, Dalla Lana School of Public Health, University of Toronto). Other members include: Mark Tyndall, MD, ScD, FRCPC (University of Ottawa); Francois Meyer, MD, PhD (Laval University); and Patrick Brown, PhD (Cancer Care Ontario, Toronto). This committee will review the accruing trial data and make recommendations regarding safety issues or reasons that may force early trial termination (e.g., early evidence of a definite efficacy due to the intervention). Members of this committee will be independent of the trial; at this point we are awaiting the IRB's recommendation with respect to the possible committee membership. Members will have expertise in statistics, HPV prevention, the conduct of clinical trials, and ethics/law. The data safety and monitoring committee will meet periodically during the course of the trial to review the data.

FINANCING AND INSURANCE

Full financing for this study has been awarded by the Canadian Institutes of Health Research (CIHR). CIHR provides funds directly to McGill University. Dr. Eduardo Franco (Principal Investigator for this study) is the Director of the Division of Cancer Epidemiology at McGill University. McGill University therefore holds the funds and maintains an award account from which Dr. Eduardo Franco can draw.

PUBLICATION POLICY

We plan to abide by the new open access publication policy recently established by CIHR. It states that "grant recipients must make every effort to ensure that their peer-reviewed research articles are freely available as soon as possible after publication." It is important to make clear that regardless of the outcome of our study, we fully intend to publish the results from our study in a journal that is accessible to vast majority of the public. CIHR suggests that this can be achieved by depositing the article in an archive, such as PubMed Central or an institutional repository, and/or by publishing results in an open access journal. One open access journal that we have considered publishing our final results is Public Library of Science (PLoS Medicine).

Table 1: Study procedures according to visit.

VISIT	0	1	2	3	4	5	6	7
	Screening Days -28 to -1	Week/Month 0/ Enrollment Visit	Week 2	Month 1	Month 3	Month 6	Month 9	Month 12/ Exit Visit
Inclusion/Exclusion Criteria	X							
Informed Consent		X						
Enrolment Questionnaire		X						
Distribution of Gels		X	X	X	X	X	X	
HPV Typing		X	X	X	X	X	X	X
AE Reporting		X	X	X	X	X	X	X
Follow-up Questionnaire			X	X	X	X	X	X
Medication Counseling		X	X	X	X	X	X	
Compliance Reinforcement		X	X	X	X	X	X	X
Assessment of Blinding						X		X

Table 2: Sample size and power calculation. [†]

Incidence Rate in Control Group (for Aim 1)	Clearance Rate in Control Group (for Aim 2)	Baseline Prevalence of HPV Infection (any type)	Anticipated Effect Size	Number of subjects required[‡] (Aim 1) Prevention	(Aim 2) Clearance
0.15	0.30	0.30	50%	634	1080
			60%	414	760
			75%	238	549
		0.40	50%	640	810
			60%	417	570
			75%	240	370
		0.50	50%	645	648
			60%	421	456
			75%	242	296
0.20	0.40	0.30	50%	455	647
			60%	297	447
			75%	171	280
		0.40	50%	459	485
			60%	299	335
			75%	173	210
		0.50	50%	463	388
			60%	302	268
			75%	174	168
0.25	0.50	0.30	50%	347	387
			60%	229	254
			75%	132	154
		0.40	50%	350	290
			60%	231	190
			75%	134	115
		0.50	50%	353	232
			60%	233	152
			75%	135	92

[†] Two-sided test at a significance level of $\alpha=0.05$ and power of 80%

[‡] Calculated sample size takes into account a 10% loss to follow-up and average baseline co-infection of 2.8 (among women testing positive for HPV at baseline), based on references 91-93

Appendix 1. Justifications for amendment

Modifying gel usage instructions to include usage after intercourse, in addition to before and during, October 9, 2015

Recent research suggests that using the carrageenan containing lubricant Divine 9 (used in CATCH) following sexual intercourse (up to two hours) in addition to using the lubricant prior to intercourse performs comparably to using the Population Council's PC-515 gel before and after intercourse. When the gels are used only before intercourse, PC-515 performs better than Divine 9 (Rodríguez et al., 2014). This suggests that using Divine 9 both before and after sexual intercourse will better protect against HPV.

We will therefore modify the instructions to include the option to apply the gel after intercourse: A women may apply the gel up to an hour before sex (current recommendation), up to two hours after sex, or both (before and after).

1) Improved protection to participants: This protocol change provides better protection to women who chose the third option, applying the gel before and after sex. Study nurses will stress to participants that applying gel before and after sex will best protect them against HPV.
2) Compliance: By adding another option for gel usage, some women who do not use the gel before or during sex may be compelled to use it afterwards. We do not have reason to believe that suggesting additional gel use will decrease compliance. We expect that women will either continue to use the gel as they have been, or use the gel more often (by using it after intercourse).
3) Enhanced scientific value to HPV epidemiology: This amendment will provide an additional variable to consider in analysis, and will further research on HPV prevention via the carrageenan containing lubricant, Divine 9.
4) Contingencies and preparedness: As a pragmatic point, CATCH has so far successfully recruited and retained only 38 individuals. This is a small fraction of the targeted sample size of 465 women. Therefore, the requested protocol amendment cannot be considered a midstream change; we are early enough in the study that a change is possible and of obvious clinical and scientific relevance.

Reference:

Rodríguez, A., Kleinbeck, K., Mizenina, et al. (2014). In vitro and in vivo evaluation of two carrageenan-based formulations to prevent HPV acquisition. *Antiviral Research*, *108*, 88–93.

Warning notice to study participants, June 12, 2017

Polyurethane condoms used by people who are allergic to latex are not compatible with the study lubricant gel, based on a report carried out by CarraShield Labs Inc. (supplier of the study gel) on the assessment of the effects of a personal lubricant on condom performance. Briefly, there is an increased risk of condom breakage with the use of the study gel with condoms made from polyurethane.

Amendment to collect 20 additional samples at enrolment, 23 May, 2019
Analyses based on the same study population that was considered in the interim analysis focused on assessing the second primary outcome of the CATCH study: clearance of HPV types that were observed at enrolment. The clearance analyses were performed on 147 HPV positive women (67 in the carrageenan group and 80 in the placebo group) out of the 280 participants. Participants in the carrageenan group who tested positive for several HPV types at enrolment were HPV negative at the second visit, but then again they tested positive at the third visit. Alarmingly, this pattern was not observed in the placebo group.
Two possible explanations for the above mentioned finding in the carrageenan group:

1- Carrageenan may have a real impact on clearance of HPV infections but needs to be applied regularly for a certain period of time to be effective. That is, the negative HPV results at the second visit after being positive at the first visit could be due to "weakened" HPV infections having a low viral load that they were undetectable. When participants ceased using the gel regularly, these infections "regained strength" and became detectable at the subsequent visit. We will be able to verify this hypothesis upon receiving the viral load results on these specimens.
2- Presence of carrageenan in vaginal samples (which is more likely to occur during the first month) may interfere with HPV detection, which if true would represent a major concern to the validity of the CATCH study. We discussed this potential inhibitory effect with our collaborator, Dr. François Coutlée, microbiologist and director of the laboratory in which HPV testing occurs. According to Dr. Coutlée, our proposed hypothesis can only be experimentally verified using real vaginal specimens.

Since recruitment is still ongoing, we will ask newly recruited participants to provide an additional self-collected vaginal specimen at enrolment (before contact with any study gel). This specimen will be combined (by the research nurse) with one of the study gels (depending on randomization allocation) before being placed in PreservCyt®. The two specimens will then be sent to Dr. Coutlée' s laboratory; both will be tested for HPV according to the usual protocol. Results should be concordant. Loss of positivity in the carrageenan swab will be evidence of inhibition of HPV detection. Since about half of the enrolled study participants tested HPV positive at baseline, we expect that 20 additional vaginal specimens (from 10 participants per study arm) will provide a sufficient number of HPV positive specimens to be paired for this comparison in each of the carrageenan and placebo groups.

REFERENCES

1. Lever Am, Berkhout B: 2008 nobel prize in medicine for discoverers of hiv. *Retrovirology* 5(1), 91 (2008).

2. Anonymous: Cheaper hpv vaccines needed. *Lancet* 371(9625), 1638 (2008).

3. Sarin R: Hpv vaccine for primary prevention of cervical cancer in developing countries: The missing links. *Journal of cancer research and therapeutics* 4(3), 105-106 (2008).

4. Hildesheim A, Herrero R, Wacholder S *et al.*: Effect of human papillomavirus 16/18 l1 viruslike particle vaccine among young women with preexisting infection: A randomized trial. *Jama* 298(7), 743-753 (2007).

5. Cuzick J, Arbyn M, Sankaranarayanan R *et al.*: Overview of human papillomavirus-based and other novel options for cervical cancer screening in developed and developing countries. *Vaccine* 26 Suppl 10, K29-41 (2008).

6. Eluf-Neto J, Nascimento Cm: Cervical cancer in latin america. *Seminars in oncology* 28(2), 188-197 (2001).

7. Parkin Dm, Bray F, Ferlay J, Pisani P: Estimating the world cancer burden: Globocan 2000. *International journal of cancer* 94(2), 153-156 (2001).

8. Franco El, Duarte-Franco E, Ferenczy A: Cervical cancer: Epidemiology, prevention and the role of human papillomavirus infection. *Cmaj* 164(7), 1017-1025 (2001).

9. Pisani P, Parkin Dm, Bray F, Ferlay J: Estimates of the worldwide mortality from 25 cancers in 1990. *International journal of cancer* 83(1), 18-29 (1999).

10. Parkin Dm, Pisani P, Ferlay J: Estimates of the worldwide incidence of 25 major cancers in 1990. *International journal of cancer* 80(6), 827-841 (1999).

11. Canadian cancer society/national cancer institute of canada: Canadian cancer statistics 2008, toronto, canada, 2008. (April 2008).

12. Schiffman Mh, Bauer Hm, Hoover Rn *et al.*: Epidemiologic evidence showing that human papillomavirus infection causes most cervical intraepithelial neoplasia. *Journal of the National Cancer Institute* 85(12), 958-964 (1993).

13. Franco El: Cancer causes revisited: Human papillomavirus and cervical neoplasia. *Journal of the National Cancer Institute* 87(11), 779-780 (1995).

14. Human papillomaviruses. *IARC monographs on the evaluation of carcinogenic risks to humans / World Health Organization, International Agency for Research on Cancer* 64, 1-378 (1995).

15. Bosch Fx, Lorincz A, Munoz N, Meijer Cj, Shah Kv: The causal relation between human papillomavirus and cervical cancer. *Journal of clinical pathology* 55(4), 244-265 (2002).

16. Monk Bj, Tewari Ks: The spectrum and clinical sequelae of human papillomavirus infection. *Gynecologic oncology* 107(2 Suppl 1), S6-13 (2007).

17. Lorincz At, Reid R, Jenson Ab, Greenberg Md, Lancaster W, Kurman Rj: Human papillomavirus infection of the cervix: Relative risk associations of 15 common anogenital types. *Obstetrics and gynecology* 79(3), 328-337 (1992).

18. Bosch Fx, Manos Mm, Munoz N *et al.*: Prevalence of human papillomavirus in cervical cancer: A worldwide perspective. International biological study on cervical cancer (ibscc) study group. *Journal of the National Cancer Institute* 87(11), 796-802 (1995).

19. Hildesheim A, Schiffman Mh, Gravitt Pe *et al.*: Persistence of type-specific human papillomavirus infection among cytologically normal women. *The Journal of infectious diseases* 169(2), 235-240 (1994).

20. Ho Gy, Bierman R, Beardsley L, Chang Cj, Burk Rd: Natural history of cervicovaginal papillomavirus infection in young women. *The New England journal of medicine* 338(7), 423-428 (1998).

21. Moscicki Ab, Shiboski S, Broering J *et al.*: The natural history of human papillomavirus infection as measured by repeated DNA testing in adolescent and young women. *The Journal of pediatrics* 132(2), 277-284 (1998).

22. Franco El, Villa Ll, Rahal P, Ruiz A: Molecular variant analysis as an epidemiological tool to study persistence of cervical human papillomavirus infection. *Journal of the National Cancer Institute* 86(20), 1558-1559 (1994).

23. Franco El, Villa Ll, Sobrinho Jp *et al.*: Epidemiology of acquisition and clearance of cervical human papillomavirus infection in women from a high-risk area for cervical cancer. *The Journal of infectious diseases* 180(5), 1415-1423 (1999).

24. Thomas Kk, Hughes Jp, Kuypers Jm *et al.*: Concurrent and sequential acquisition of different genital human papillomavirus types. *The Journal of infectious diseases* 182(4), 1097-1102 (2000).

25. Liaw Kl, Hildesheim A, Burk Rd *et al.*: A prospective study of human papillomavirus (hpv) type 16 DNA detection by polymerase chain reaction and its association with acquisition and persistence of other hpv types. *The Journal of infectious diseases* 183(1), 8-15 (2001).

26. Ho Gy, Burk Rd, Klein S *et al.*: Persistent genital human papillomavirus infection as a risk factor for persistent cervical dysplasia. *Journal of the National Cancer Institute* 87(18), 1365-1371 (1995).

27. Remmink Aj, Walboomers Jm, Helmerhorst Tj *et al.*: The presence of persistent high-risk hpv genotypes in dysplastic cervical lesions is associated with progressive disease: Natural history up to 36 months. *International journal of cancer* 61(3), 306-311 (1995).

28. Liaw Kl, Glass Ag, Manos Mm *et al.*: Detection of human papillomavirus DNA in cytologically normal women and subsequent cervical squamous intraepithelial lesions. *Journal of the National Cancer Institute* 91(11), 954-960 (1999).

29. Ylitalo N, Josefsson A, Melbye M *et al.*: A prospective study showing long-term infection with human papillomavirus 16 before the development of cervical carcinoma in situ. *Cancer research* 60(21), 6027-6032 (2000).

30. Cates W, Jr., Stone Km: Family planning, sexually transmitted diseases and contraceptive choice: A literature update--part i. *Family planning perspectives* 24(2), 75-84 (1992).

31. Stratton P, Alexander Nj: Prevention of sexually transmitted infections. Physical and chemical barrier methods. *Infectious disease clinics of North America* 7(4), 841-859 (1993).

32. Manhart Le, Koutsky La: Do condoms prevent genital hpv infection, external genital warts, or cervical neoplasia? A meta-analysis. *Sexually transmitted diseases* 29(11), 725-735 (2002).

33. Franco El: Understanding the epidemiology of genital infection with oncogenic and nononcogenic human papillomaviruses: A promising lead for primary prevention of cervical cancer. *Cancer Epidemiol Biomarkers Prev* 6(10), 759-761 (1997).

34. Richardson H, Franco E, Pintos J, Bergeron J, Arella M, Tellier P: Determinants of low-risk and high-risk cervical human papillomavirus infections in montreal university students. *Sexually transmitted diseases* 27(2), 79-86 (2000).

35. Aral So, Peterman Ta: A stratified approach to untangling the behavioral/biomedical outcomes conundrum. *Sexually transmitted diseases* 29(9), 530-532 (2002).

36. Macaluso M, Demand Mj, Artz Lm, Hook Ew, 3rd: Partner type and condom use. *AIDS (London, England)* 14(5), 537-546 (2000).

37. Warner L, Newman Dr, Austin Hd *et al.*: Condom effectiveness for reducing transmission of gonorrhea and chlamydia: The importance of assessing partner infection status. *American journal of epidemiology* 159(3), 242-251 (2004).

38. Winer Rl, Hughes Jp, Feng Q *et al.*: Condom use and the risk of genital human papillomavirus infection in young women. *The New England journal of medicine* 354(25), 2645-2654 (2006).

39. The Future Ii Study Group: Quadrivalent vaccine against human papillomavirus to prevent high-grade cervical lesions. *The New England journal of medicine* 356(19), 1915-1927 (2007).

40. Harper Dm, Franco El, Wheeler Cm *et al.*: Sustained efficacy up to 4.5 years of a bivalent l1 virus-like particle vaccine against human papillomavirus types 16 and 18: Follow-up from a randomised control trial. *Lancet* 367(9518), 1247-1255 (2006).

41. Ault Ka: Effect of prophylactic human papillomavirus l1 virus-like-particle vaccine on risk of cervical intraepithelial neoplasia grade 2, grade 3, and adenocarcinoma in situ: A combined analysis of four randomised clinical trials. *Lancet* 369(9576), 1861-1868 (2007).

42. Garland Sm, Hernandez-Avila M, Wheeler Cm *et al.*: Quadrivalent vaccine against human papillomavirus to prevent anogenital diseases. *The New England journal of medicine* 356(19), 1928-1943 (2007).

43. Schiffman M, Castle Pe, Jeronimo J, Rodriguez Ac, Wacholder S: Human papillomavirus and cervical cancer. *Lancet* 370(9590), 890-907 (2007).

44. Harper Dm, Franco El, Wheeler C *et al.*: Efficacy of a bivalent l1 virus-like particle vaccine in prevention of infection with human papillomavirus types 16 and 18 in young women: A randomised controlled trial. *Lancet* 364(9447), 1757-1765 (2004).

45. Adams M, Jasani B, Fiander A: Human papilloma virus (hpv) prophylactic vaccination: Challenges for public health and implications for screening. *Vaccine* 25(16), 3007-3013 (2007).

46. Brisson M, Van De Velde N, De Wals P, Boily Mc: The potential cost-effectiveness of prophylactic human papillomavirus vaccines in canada. *Vaccine* 25(29), 5399-5408 (2007).

47. Kim Jj, Goldie Sj: Health and economic implications of hpv vaccination in the united states. *The New England journal of medicine* 359(8), 821-832 (2008).

48. Eichler Hg, Kong Sx, Gerth Wc, Mavros P, Jonsson B: Use of cost-effectiveness analysis in health-care resource allocation decision-making: How are cost-effectiveness thresholds expected to emerge? *Value Health* 7(5), 518-528 (2004).

49. Franco El, Ferenczy A: Cervical cancer screening following the implementation of prophylactic human papillomavirus vaccination. *Future oncology (London, England)* 3(3), 319-327 (2007).

50. Goldie Sj, Kohli M, Grima D *et al.*: Projected clinical benefits and cost-effectiveness of a human papillomavirus 16/18 vaccine. *Journal of the National Cancer Institute* 96(8), 604-615 (2004).

51. Munoz N, Bosch Fx, Castellsague X *et al.*: Against which human papillomavirus types shall we vaccinate and screen? The international perspective. *International journal of cancer* 111(2), 278-285 (2004).

52. Dunne Ef, Unger Er, Sternberg M *et al.*: Prevalence of hpv infection among females in the united states. *Jama* 297(8), 813-819 (2007).

53. De Sanjose S, Diaz M, Castellsague X *et al.*: Worldwide prevalence and genotype distribution of cervical human papillomavirus DNA in women with normal cytology: A meta-analysis. *The Lancet infectious diseases* 7(7), 453-459 (2007).

54. Garland Sm, Brotherton Jm, Skinner Sr *et al.*: Human papillomavirus and cervical cancer in australasia and oceania: Risk-factors, epidemiology and prevention. *Vaccine* 26 Suppl 12, M80-88 (2008).

55. Buck Cb, Thompson Cd, Roberts Jn, Muller M, Lowy Dr, Schiller Jt: Carrageenan is a potent inhibitor of papillomavirus infection. *PLoS pathogens* 2(7), e69 (2006).

56. Bagchi S: Red-algae derivative could be useful adjunct to hpv vaccine. *The lancet oncology* 7(8), 623 (2006).

57. Roberts Jn, Buck Cb, Thompson Cd *et al.*: Genital transmission of hpv in a mouse model is potentiated by nonoxynol-9 and inhibited by carrageenan. *Nature medicine* 13(7), 857-861 (2007).

58. Munoz N, Bosch Fx, De Sanjose S *et al.*: Epidemiologic classification of human papillomavirus types associated with cervical cancer. *The New England journal of medicine* 348(6), 518-527 (2003).

59. Freedman B: Equipoise and the ethics of clinical research. *The New England journal of medicine* 317(3), 141-145 (1987).

60. Howett Mk, Kuhl Jp: Microbicides for prevention of transmission of sexually transmitted diseases. *Current pharmaceutical design* 11(29), 3731-3746 (2005).

61. Chan Pj, Su Bc, Kalugdan T, Seraj Im, Tredway Dr, King A: Human papillomavirus gene sequences in washed human sperm deoxyribonucleic acid. *Fertility and sterility* 61(5), 982-985 (1994).

62. Lai Ym, Yang Fp, Pao Cc: Human papillomavirus deoxyribonucleic acid and ribonucleic acid in seminal plasma and sperm cells. *Fertility and sterility* 65(5), 1026-1030 (1996).

63. Lai Ym, Lee Jf, Huang Hy, Soong Yk, Yang Fp, Pao Cc: The effect of human papillomavirus infection on sperm cell motility. *Fertility and sterility* 67(6), 1152-1155 (1997).

64. Olatunbosun Oa, Case Am, Deneer Hg: Detection of human papillomavirus DNA in sperm using polymerase chain reaction. *Methods in molecular biology (Clifton, N.J* 253, 95-104 (2004).

65. Foresta C, Garolla A, Zuccarello D *et al.*: Human papillomavirus found in sperm head of young adult males affects the progressive motility. *Fertility and sterility*, (2008).

66. Perez-Andino J, Buck Cb, Ribbeck K: Adsorption of human papillomavirus 16 to live human sperm. *PLoS ONE* 4(6), e5847 (2009).

67. Van De Wijgert J, Jones H, Pistorius A *et al.*: Phase iii microbicide trial methodology: Opinions of experienced expanded safety trial participants in south africa. *Sahara J* 2(3), 311-319 (2005).

68. Skoler-Karpoff S, Ramjee G, Ahmed K *et al.*: Efficacy of carraguard for prevention of hiv infection in women in south africa: A randomised, double-blind, placebo-controlled trial. *Lancet* 372(9654), 1977-1987 (2008).

69. Coggins C, Blanchard K, Alvarez F *et al.*: Preliminary safety and acceptability of a carrageenan gel for possible use as a vaginal microbicide. *Sexually transmitted infections* 76(6), 480-483 (2000).

70. Ramjee G, Morar Ns, Braunstein S, Friedland B, Jones H, Van De Wijgert J: Acceptability of carraguard, a candidate microbicide and methyl cellulose placebo vaginal gels among hiv-positive women and men in durban, south africa. *AIDS research and therapy* 4, 20 (2007).

71. Elias Cj, Coggins C, Alvarez F *et al.*: Colposcopic evaluation of a vaginal gel formulation of iota-carrageenan. *Contraception* 56(6), 387-389 (1997).

72. Kilmarx Ph, Van De Wijgert Jh, Chaikummao S *et al.*: Safety and acceptability of the candidate microbicide carraguard in thai women: Findings from a phase ii clinical trial. *Journal of acquired immune deficiency syndromes (1999)* 43(3), 327-334 (2006).

73. Kilmarx Ph, Blanchard K, Chaikummao S *et al.*: A randomized, placebo-controlled trial to assess the safety and acceptability of use of carraguard vaginal gel by heterosexual couples in thailand. *Sexually transmitted diseases* 35(3), 226-232 (2008).

74. Van De Wijgert Jh, Braunstein Sl, Morar Ns *et al.*: Carraguard vaginal gel safety in hiv-positive women and men in south africa. *Journal of acquired immune deficiency syndromes (1999)* 46(5), 538-546 (2007).

75. Kreuzer M: Spreading the message: The significance of ce-marking. *Medical device technology* 9(4), 38-39 (1998).

76. Rousseau Mc, Pereira Js, Prado Jc, Villa Ll, Rohan Te, Franco El: Cervical coinfection with human papillomavirus (hpv) types as a predictor of acquisition and persistence of hpv infection. *The Journal of infectious diseases* 184(12), 1508-1517 (2001).

77. Bauer Hm, Hildesheim A, Schiffman Mh *et al.*: Determinants of genital human papillomavirus infection in low-risk women in portland, oregon. *Sexually transmitted diseases* 20(5), 274-278 (1993).

78. Wheeler Cm, Parmenter Ca, Hunt Wc *et al.*: Determinants of genital human papillomavirus infection among cytologically normal women attending the university of new mexico student health center. *Sexually transmitted diseases* 20(5), 286-289 (1993).

79. Burk Rd, Ho Gy, Beardsley L, Lempa M, Peters M, Bierman R: Sexual behavior and partner characteristics are the predominant risk factors for genital human papillomavirus infection in young women. *The Journal of infectious diseases* 174(4), 679-689 (1996).

80. Franco El, Villa Ll, Ruiz A, Costa Mc: Transmission of cervical human papillomavirus infection by sexual activity: Differences between low and high oncogenic risk types. *The Journal of infectious diseases* 172(3), 756-763 (1995).

81. Collins S, Mazloomzadeh S, Winter H *et al.*: High incidence of cervical human papillomavirus infection in women during their first sexual relationship. *Bjog* 109(1), 96-98 (2002).

82. Centers for disease control national immunization survey report. (2008, posting date).

83. Saslow D, Castle Pe, Cox Jt *et al.*: American cancer society guideline for human papillomavirus (hpv) vaccine use to prevent cervical cancer and its precursors. *CA: a cancer journal for clinicians* 57(1), 7-28 (2007).

84. Macaluso M, Lawson Ml, Hortin G *et al.*: Efficacy of the female condom as a barrier to semen during intercourse. *American journal of epidemiology* 157(4), 289-297 (2003).

85. Wright Tc, Jr., Denny L, Kuhn L, Pollack A, Lorincz A: Hpv DNA testing of self-collected vaginal samples compared with cytologic screening to detect cervical cancer. *Jama* 283(1), 81-86 (2000).

86. Sellors Jw, Lorincz At, Mahony Jb *et al.*: Comparison of self-collected vaginal, vulvar and urine samples with physician-collected cervical samples for human papillomavirus testing to detect high-grade squamous intraepithelial lesions. *Cmaj* 163(5), 513-518 (2000).

87. Gravitt Pe, Lacey Jv, Jr., Brinton La *et al.*: Evaluation of self-collected cervicovaginal cell samples for human papillomavirus testing by polymerase chain reaction. *Cancer Epidemiol Biomarkers Prev* 10(2), 95-100 (2001).

88. Tarkowski Ta, Rajeevan Ms, Lee Dr, Unger Er: Improved detection of viral rna isolated from liquid-based cytology samples. *Mol Diagn* 6(2), 125-130 (2001).

89. Coutlee F, Rouleau D, Petignat P *et al.*: Enhanced detection and typing of human papillomavirus (hpv) DNA in anogenital samples with pgmy primers and the linear array hpv genotyping test. *Journal of clinical microbiology* 44(6), 1998-2006 (2006).

90. Clarke P, Ebel C, Catotti Dn, Stewart S: The psychosocial impact of human papillomavirus infection: Implications for health care providers. *International journal of STD & AIDS* 7(3), 197-200 (1996).

91. Richardson H, Kelsall G, Tellier P *et al.*: The natural history of type-specific human papillomavirus infections in female university students. *Cancer Epidemiol Biomarkers Prev* 12(6), 485-490 (2003).

92. Burchell An, Tellier Pp, Hanley J, Coutlee F, Franco El: Influence of partner's infection status on prevalent human papillomavirus among persons with a new sex partner. *Sexually transmitted diseases*, (2009).

93. Burchell An, Tellier Pp, Hanley J, Coutlee F, Franco El: Human papillomavirus infections among couples in new sexual relationships. *Epidemiology* 21(1), 31-37

94. Dupont Wd, Plummer Wd, Jr.: Power and sample size calculations. A review and computer program. *Controlled clinical trials* 11(2), 116-128 (1990).

95. Burchell An, Richardson H, Mahmud Sm *et al.*: Modeling the sexual transmissibility of human papillomavirus infection using stochastic computer simulation and empirical data from a cohort study of young women in montreal, canada. *American journal of epidemiology* 163(6), 534-543 (2006).

96. Richardson H, Abrahamowicz M, Tellier Pp *et al.*: Modifiable risk factors associated with clearance of type-specific cervical human papillomavirus infections in a cohort of university students. *Cancer Epidemiol Biomarkers Prev* 14(5), 1149-1156 (2005).

97. Cohen J: Aids research. Microbicide fails to protect against hiv. *Science (New York, N.Y* 319(5866), 1026-1027 (2008).

98. Behets Fm, Turner An, Van Damme K *et al.*: Vaginal microbicide and diaphragm use for sexually transmitted infection prevention: A randomized acceptability and feasibility study among high-risk women in madagascar. *Sexually transmitted diseases* 35(9), 818-826 (2008).

99. Feldblum Pj, Adeiga A, Bakare R *et al.*: Savvy vaginal gel (c31g) for prevention of hiv infection: A randomized controlled trial in nigeria. *PLoS ONE* 3(1), e1474 (2008).

100. Short Mb, Succop Pa, Ugueto Am, Rosenthal Sl: Predictors of using a microbicide-like product among adolescent girls. *J Adolesc Health* 41(4), 357-362 (2007).

101. Graham Ca, Crosby Ra, Sanders Sa, Yarber Wl: Assessment of condom use in men and women. *Annual review of sex research* 16, 20-52 (2005).

102. Shew Ml, Remafedi Gj, Bearinger Lh *et al.*: The validity of self-reported condom use among adolescents. *Sexually transmitted diseases* 24(9), 503-510 (1997).

103. Cecil H, Zimet Gd: Meanings assigned by undergraduates to frequency statements of condom use. *Archives of sexual behavior* 27(5), 493-505 (1998).

104. Lan Kk, Rosenberger Wf, Lachin Jm: Use of spending functions for occasional or continuous monitoring of data in clinical trials. *Statistics in medicine* 12(23), 2219-2231 (1993).

105. O'brien Pc, Fleming Tr: A multiple testing procedure for clinical trials. *Biometrics* 35(3), 549-556 (1979).

106. Winer Rl, Lee Sk, Hughes Jp, Adam De, Kiviat Nb, Koutsky La: Genital human papillomavirus infection: Incidence and risk factors in a cohort of female university students. *American journal of epidemiology* 157(3), 218-226 (2003).

107. De Camargo B, Franco El: Single-dose versus fractionated-dose dactinomycin in the treatment of wilms' tumor. Preliminary results of a clinical trial. The brazilian wilms' tumor study group. *Cancer* 67(12), 2990-2996 (1991).

108. De Camargo B, Franco El: A randomized clinical trial of single-dose versus fractionated-dose dactinomycin in the treatment of wilms' tumor. Results after extended follow-up. Brazilian wilms' tumor study group. *Cancer* 73(12), 3081-3086 (1994).

109. Payment P, Richardson L, Siemiatycki J, Dewar R, Edwardes M, Franco E: A randomized trial to evaluate the risk of gastrointestinal disease due to consumption of drinking water meeting current microbiological standards. *American journal of public health* 81(6), 703-708 (1991).

110. Mayrand Mh, Duarte-Franco E, Coutlee F *et al.*: Randomized controlled trial of human papillomavirus testing versus pap cytology in the primary screening for cervical cancer precursors: Design, methods and preliminary accrual results of the canadian cervical cancer screening trial (cccast). *International journal of cancer* 119(3), 615-623 (2006).

111. Mayrand Mh, Duarte-Franco E, Rodrigues I *et al.*: Human papillomavirus DNA versus papanicolaou screening tests for cervical cancer. *The New England journal of medicine* 357(16), 1579-1588 (2007).
